# Isolation of Low Dispersity Fractions of Acetone Organosolv Lignins to Understand their Reactivity: Towards Aromatic Building Blocks for Polymers Synthesis

**DOI:** 10.1002/cssc.202001976

**Published:** 2020-10-16

**Authors:** Antoine Duval, Géraldine Layrac, André van Zomeren, Arjan T. Smit, Eric Pollet, Luc Avérous

**Affiliations:** ^1^ BioTeam/ICPEES-ECPM UMR CNRS 7515 Université de Strasbourg 25 rue Becquerel 67087 Strasbourg Cedex 2 France; ^2^ TNO-Energy Transition Westerduinweg 3 1755 LE Petten (The Netherlands

**Keywords:** ethylene carbonate, fractionation, lignin, organosolv, solubility

## Abstract

Two organosolv lignins extracted during pilot runs of the Fabiola process were analyzed, fractionated and chemically modified with ethylene carbonate (EC) to produce building blocks suitable for polymer synthesis. Isolation of low dispersity fractions relied on the partial solubility of the lignins in organic solvents. Lignins solubility was first evaluated and analyzed with Hansen and Kamlet‐Taft solubility parameters, showing a good correlation with the solvents dipolarity/polarizability parameter π*. The results were then used to select a sequence of solvents able to fractionate the lignins into low dispersity fractions of increasing molar masses, which were analyzed by ^31^P NMR, SEC and DSC. The lignins were then reacted with EC, to convert the phenolic OH groups into primary aliphatic OH groups. The reactivity of the organosolv lignins was high, and milder reaction conditions than previously reported were sufficient to fully convert the phenolic OH groups. A gradual reduction in reactivity with increasing molar mass was evidenced and attributed to reduced solubility of high molar mass fragments in EC. Undesirable crosslinking side reactions were evidenced by SEC, but were efficiently limited thanks to a fine control of the reaction conditions, helping to maximize the benefits of the developed lignin modification with EC.

## Graphical abstract

**EC does it**: Organosolv FABIOLA lignins are characterized, fractionated and modified with ethylene carbonate to produce aromatic building blocks exposing primary aliphatic OH groups that can be used in polymer synthesis.

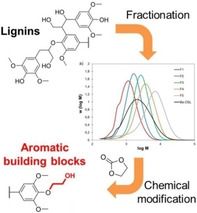

## Introduction

The need to reduce greenhouse gases emissions is a driver for the development of renewable feedstocks for production of energy, chemicals and materials. Because of its worldwide availability in large volumes and non‐competition with food supply, lignocellulosic biomass appears as the feedstock of choice. It is mainly composed of cellulose, hemicellulose and lignin, which are intimately associated within the plant cell walls. Lignocellulosic biorefineries aim at fractionating and refining the biomass into different streams, which can serve various applications. Organosolv pretreatments have proven to be among the most promising for such purpose, and recent years have seen considerable developments.[^1^, ^2^, ^3^, ^4^, ^5^, ^6^] The efficient use of all the individual constituents of lignocellulosic biomass is crucial for the economic viability of biorefineries, and has raised increased attention to the valorization of lignins.

Lignins constitute the main renewable source of aromatic structures, which are known to enhance the stability, mechanical and thermal properties of polymers. Many studies have thus focused on the use of lignin as component of polymer materials in the past decade.[^7^, ^8^, ^9^] A large part of the developed polymeric systems relies on the reactivity of lignin chemical functions, particularly phenolic OH groups. For several applications, such as the synthesis of polyesters or polyurethanes, the reactivity of the phenolic OH groups is rather limited. Then, the conversion of the phenolic OH groups of lignin into more reactive aliphatic OH groups, which has been investigated for a long time,[^10^, ^11^, ^12^, ^13^, ^14^] seems to be a relevant approach.

The modification of polyphenols, including various lignins, with cyclic carbonates has been recently developed in our laboratory.[^15^, ^16^, ^17^] This method is particularly attractive with the simplest cyclic carbonate, ethylene carbonate (EC), since it allows the introduction of primary aliphatic OH groups of high reactivity on the lignin. Such lignins modified with EC can indeed be directly esterified by fatty acids in the absence of catalyst or solvent.[^18^, ^19^] They also show a higher reactivity towards isocyanates and can thus be employed in the synthesis of polyurethane foams.^[20]^

Recently, a mild acetone organosolv fractionation process was developed and successfully applied to various hardwoods and herbaceous biomass.^[21]^ Pilot runs of the so‐called Fabiola™ process have been performed with two hardwoods, beech and birch. This study aims at examining the properties of the isolated lignins, and their suitability for further chemical reactions towards applications in polymer materials. To get better insights into the lignin reactivity, a solvent‐based fractionation into low dispersity fractions was performed, following a method previously developed for Kraft lignin.^[22]^ Fractionation of lignin allows to obtain well‐defined fractions of low dispersity and more uniform structure, thus potentially improving their use in high value applications. The past few years have seen considerable developments in this area, and recent reviews give an exhaustive overview of the different methods that have been used.[^23^, ^24^, ^25^]

A detailed study of the solubility in common organic solvents was first performed to find the best conditions to fractionate the organosolv lignins from beech and birch. Both lignins were then refined into five distinct fractions, which were characterized by ^31^P NMR, FTIR spectroscopy, size exclusion chromatography (SEC) and differential scanning calorimetry (DSC). The lignins were then modified with EC, with a particular emphasis on the search of mild reaction conditions able to limit side reactions such as crosslinking,^[26]^ which can be detrimental to the properties of the modified lignins. The previously isolated fractions were also reacted with EC to gain insights into the influence of lignin molar mass and functional groups content on the reactivity.

## Results and Discussion

### Solubility of Fabiola lignins in organic solvents

To determine which solvents would be suitable for the fractionation of the organosolv lignins from beech and birch, solubility tests in various organic solvents were performed. The choice of the tested solvents was based on a literature survey of preexisting procedures for lignin fractionation, as well as on safety and environmental considerations. Based on recent solvent selection guides,[^27^, ^28^, ^29^, ^30^, ^31^] we limited our choice to solvents ranked as recommended, leading to the exclusion of solvents commonly used for lignin fractionation, such as dichloromethane[^32^, ^33^, ^34^] or diethyl ether.[^35^, ^36^, ^37^] The selected solvents were thus alcohols (methanol, ethanol, 1‐propanol and isopropanol), ketones (acetone and methyl ethyl ketone) and an ester (ethyl acetate).

**Figure 1:**
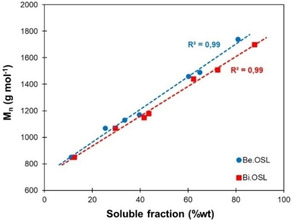
Average molar mass of the soluble fractions depending on their solubility yield.

Both beech and birch lignins were partially soluble in all the tested solvents, and the differences between beech and birch lignins were only marginal. Table 1 shows the results of the solubility tests, together with the molar masses of the soluble and insoluble fractions determined by SEC. The corresponding chromatograms are available in the Supporting Information (Figures S1 and S2). For a given solvent, the average between the *M_w_* of the soluble and insoluble fractions is equal to the initial molar mass of the unfractionated lignin within a ±5% margin. This shows that the solubilization process does not induce any depolymerization or aggregation of the lignins that would lead to apparently lower or higher molar masses, respectively. The results show that the average molar mass of the soluble fractions increases linearly with the solubility (Figure 1). Low molar mass fragments are easily dissolved in a wide variety of solvents, whereas higher molar mass fragments are more complex to solubilize and require solvents with increased affinity. This specific variation in solubility depending on the solvent nature has then been exploited to develop a sequential solvent fractionation (see below).

**Table 1:** Yields (in wt %), average molar masses and dispersities of the soluble and insoluble fractions of beech and birch lignins in various organic solvents. SEC distributions are available in the Supporting Information.

Lignin	Solvent	Soluble fraction				Insoluble fraction			
		[wt %]	*M*_n_ [g mol^−1^]	*M*_w_ [g mol^−1^]	*Đ*	[wt %]	*M*_n_ [g mol^−1^]	*M*_w_ [g mol^−1^]	*Đ*
Be.OSL	–	–	1810	3610	1.99	–	–	–	–
	isopropanol	11.1	850	1230	1.45	87.7	2060	3790	1.84
	1‐propanol	25.3	1070	1550	1.45	70.7	2470	4240	1.72
	ethyl acetate	33.5	1130	1600	1.42	66.8	2890	4750	1.64
	ethanol	39.6	1170	1770	1.51	60.2	2910	4770	1.64
	methanol	60.1	1460	2410	1.65	36.7	3050	5640	1.79
	methyl ethyl ketone	64.9	1490	2640	1.77	37.9	2920	7910	2.47
	acetone	80.8	1740	3260	1.87	17.7	3350	6930	2.07
Bi.OSL	–	–	1760	3440	1.95	–	–	–	–
	isopropanol	12.3	850	1120	1.32	85.2	2110	3870	1.83
	1‐propanol	29.5	1070	1590	1.49	66.6	2350	3930	1.67
	ethyl acetate	41.6	1150	1640	1.43	60.2	2910	4720	1.62
	ethanol	43.6	1180	1770	1.50	57.0	2830	4600	1.63
	methanol	62.2	1440	2360	1.64	35.5	2670	5060	1.90
	methyl ethyl ketone	72.3	1510	2710	1.79	30.3	2780	7220	2.60
	acetone	87.7	1700	3290	1.94	11.8	3350	7020	2.10

The solubility of the lignins was then analyzed with respect to different theoretical solubility models. In addition to the solvents tested earlier, three solvents able to fully dissolve both lignins were also included as reference (DMSO, DMF and pyridine). The solubility parameter *δ* was first introduced by Hildebrand and Scott^[38]^ as [Eq. 1]:

(1) $$\delta =\;\sqrt{\frac{E}{V_{m}}}$$

where *E* is the energy of vaporization and *V*_m_ is the molar volume of the solvent.

In this theory, a solute is likely to be dissolved by a solvent having a *δ* close to its own. This approach was then extended by Hansen,^[39]^ considering that the energy of vaporization is the resultant of different contributions. This led him to introduce the now called Hansen solubility parameters (HSPs), related to the solvents dispersion forces (*δ_D_*), polar interactions (*δ_P_*) and hydrogen bonding ability (*δ_H_*), which are related to Hildebrand parameter by Equation 2:^[39]^

(2) $$\delta =\;\sqrt{\delta_{P}^{2}+\;\delta_{D}^{2}+\;\delta_{H}^{2}}$$

**Figure 2:**
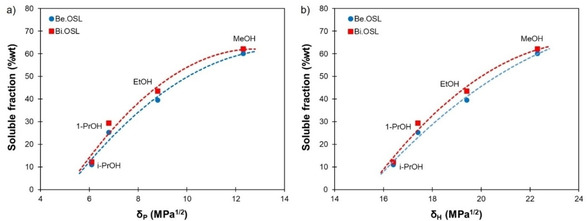
Solubility of beech and birch lignins in alcohols depending on their Hansen solubility parameters: (a) polarity δ_P_ and (b) hydrogen bonding ability δ_H_.

The solubility of the lignins in alcohols was found to be well correlated with their *δ*_P_ and *δ*_H_ (Figure 2). Increasing the solvent polarity and hydrogen bonding ability leads to a better dissolution of lignin, in agreement with earlier reports.[^22^, ^40^, ^41^] Similar correlations within other groups of solvents, such as ketones or esters, could also exist but would require additional experiments with a larger set of solvents. However, when considering all the tested solvents together, no correlations could be evidenced with any of the individual HSP nor *δ* (Supporting Information, Figure S3). In Hansen's theoretical approach, HSPs should be considered together rather individually since they all play a synergistic role in solubility. The “distance” between a solvent of HSPs (*δ*_D1_, *δ*_P1_, *δ*_H1_), and a solute of HSPs (*δ*_D2_, *δ*_P2_, *δ*_H2_), is calculated by Equation 3:^[39]^

(3) $$R_{a}=\;\sqrt{4\;{\left(\delta_{D1}-\;\delta_{D2}\right)}^{2}+{\left(\delta_{P1}-\;\delta_{P2}\right)}^{2}+{\left(\delta_{H1}-\;\delta_{H2}\right)}^{2}}$$

Solubility should be achieved for *R*_a_<*R*_0_, where R_0_ is a fourth parameter characteristic of the solute,^[39]^ or when [Eq. 4]:

(4) $$RED=\;\frac{R_{a}}{R_{0}}<1$$

where RED stands for relative energy difference.

To calculate the *RED* between the lignins and the tested solvents, it is necessary to know the HSPs of lignins. Several values from the literature[^42^, ^43^, ^44^] were used to calculate the *RED*, and the results are presented in the Supporting Information (Figure S4). However, they do not show any correlation with the solubility, as already experienced previously with softwood Kraft lignin.^[22]^ The reported HSPs of lignin were calculated for softwood^[42]^ and sugarcane bagasse lignin,^[44]^ respectively. Given the structural differences between lignins from various botanical origins and extraction processes, significant differences in HSPs might be expected. This can explain the impossibility to accurately describe the solubility of the hardwood organosolv lignins studied here with HSPs.

**Figure 3:**
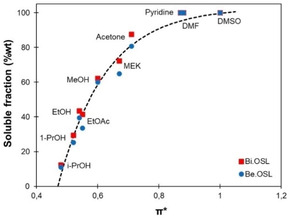
Solubility of beech and birch lignins depending on the solvent polarity/polarizability parameter π*.

The results of the solubility tests were further analyzed considering the Kamlet‐Taft solvatochromic parameters.^[45]^ In this approach, the solvents are characterized by three parameters, describing their dipolarity/polarizability (π*) and their ability to donate (α) or accept hydrogen bonds (β). Many chemical or physico‐chemical properties, including solubility, have been shown to depend on one or more of the solvent parameters via the so‐called Linear Solvation Energy Relationship (LSER).^[46]^ Plots of the solubility against α or β do not show any correlation (Supporting Information, Figure S5). Interestingly, there is a good correlation with π*, as depicted on Figure 3. An increase in the solvent dipolarity/polarizability parameter leads to a better dissolution of the lignin. For π*≥0.87, full solubility is achieved. This is to the best of our knowledge the first time that such a correlation between lignin solubility and Kamlet‐Taft solubility parameters is evidenced. Another interesting observation is that the correlation seems to be valid for both the birch and the beech lignin. Although further testing of a wider range of lignin sources is needed, this observation might imply that the found correlation is more generally applicable to lignins.

### Sequential solvent fractionation: towards low dispersity lignin fractions

**Figure 4:**
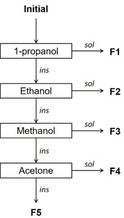
Scheme of the sequential solvent fractionation applied to beech and birch lignins.

Based on the results of the solubility tests, 4 solvents were selected to isolate 5 different fractions, with the objective to obtain equilibrated yields, between 15 and 25 wt%. Since the yields of the different fractions can be estimated from the solubility tests (Supporting Information),[^22^, ^47^] the solvent sequence presented on Figure 4 was adopted. As compared to the solvent sequence previously adopted for softwood Kraft lignin,^[22]^ only ethyl acetate was replaced by 1‐propanol.

**Figure 5:**
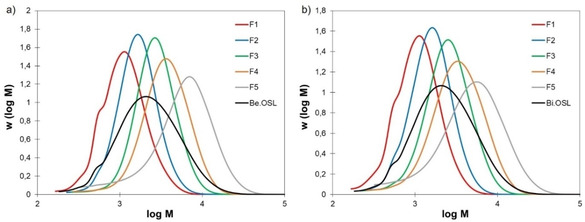
SEC distributions of the initial lignins and the fractions isolated from (a) beech and (b) birch lignins.

The experimental yields of the different fractions were well correlated to the predicted yields (Supporting Information, Figure S6). For beech lignin, they ranged from 16.6 to 24.0 wt%, whereas they were between 14.0 and 25.8 wt% for birch lignin (Table S3). Analysis of the fractions by SEC confirmed the success of the fractionation steps. The fractions have increasing molar mass from F1 to F5 and low dispersity (Figure 5 and Table S3). Molar mass differences between beech and birch lignin fractions were minimal.

**Figure 6:**
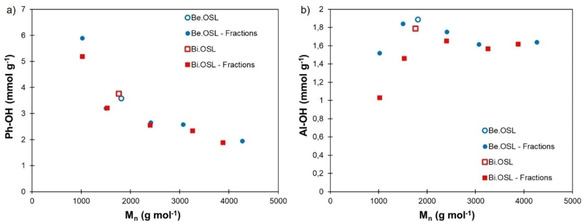
Content in OH groups of beech and birch lignins and their fractions depending on the average molar mass: (a) phenolic OH, (b) aliphatic OH.

The lignins were then analyzed by a modified Klason method,^[48]^ which showed good purity with a total lignin content higher than 93 wt% (Table S4). After fractionation, the low molar mass fractions F1 have significantly lower content in acid insoluble lignin and higher content in acid soluble lignin than the other fractions. The content in functional groups of the lignin fractions was determined by ^31^P NMR. The spectra are available in the Supporting Information (Figures S7 and S8). Both lignins present the expected characteristics of hardwood lignins, with the predominance of syringyl (S) units, the presence of guaiacyl (G) units and absence of *p*‐hydroxyphenyl (H) units. The amount of phenolic OH groups present on the lignin fractions was found to correlate with the average molar mass (Figure 6a). Indeed, phenolic OH groups are chain ends of the lignin oligomers, which explains that their concentration increases when the average molar mass decreases. Aliphatic OH groups are distributed more evenly among the lignin fractions (Figure 6b), with only the low molar mass fractions containing significantly less than the others. Similar trends have been reported on different kind of lignins, such as softwood[^34^, ^47^, ^49^, ^50^, ^51^] and hardwood Kraft lignins,[^47^, ^49^, ^52^] soda lignins[^53^, ^54^, ^55^] or various organosolv lignins from hardwood[^35^, ^56^] or annual plants.^[57]^ Both beech and birch lignins contain very few carboxylic acid groups (less than 0.1 mmol g^−1^), which are all found in the low molar mass fractions F1 (Supporting Information, Table S3, Figures S7 and S8).

The fractionation was then scaled up to 50 g of beech lignin. The yields of the different fractions were slightly different from the fractionation at smaller scale (Supporting Information, Figure S9). The yields of the two first fractions were lower, whereas the yield of the last fraction F5 was higher. The differences can come from the difficulty to achieve a homogeneous stirring of the lignin suspension, which can lead to a reduce solubilization in the solvents with the lowest ability to dissolve the lignins. However, the molar mass distributions are similar (Supporting Information, Figure S10), indicating that the developed solvent fractionation is suitable at this scale.

**Figure 7:**
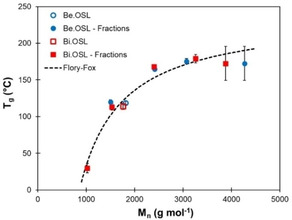
Glass transition temperature (T_g_) of beech and birch lignins and their fractions depending on the average molar mass, and fit of the data according to the Flory‐Fox equation.

The glass transition temperatures (*T*_g_) of the lignin fractions were determined by DSC (DSC curves are available in the Supporting Information, Figures S11 and S12). As expected, *T_g_* increases with the molar mass, before to reach a plateau at about 170 °C, as shown in Figure 7. High standard deviations were observed for the fractions F5, as a result of their high dispersity (Figure 5). The data were tentatively fitted to the Flory‐Fox relationship:^[58]^

(5) $$T_{g}=\;T_{g}^{\infty }-\;\frac{K}{M_{n}}$$

where *T*_g_^*∞*^ is the *T*_g_ of a theoretical infinite molar mass polymer and K is a constant.

The quality of the fit is relatively good (R^2^=0.95, Supporting Information, Figure S13), although in branched polymers like lignins, several other parameters than the molar mass have been reported to influence the *T*_g_, such as the degree of branching[^59^, ^60^, ^61^] or the nature of the terminal groups.^[62]^

### Modification of lignins with ethylene carbonate (EC)

**Scheme 1:**
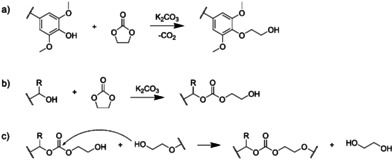
Reaction of lignins with ethylene carbonate (a) formation of ether linkages on the phenolic OH groups, (b) formation of carbonate linkages on the aliphatic OH groups, (c) possible crosslinking side reactions by transcarbonation, leading to the formation of high molar mass fragments.

Beech and birch lignins were then reacted with ethylene carbonate (EC). This reaction, depicted on Scheme 1, has been developed by some of us as a powerful method to convert the phenolic OH of lignins into primary aliphatic OH (Scheme 1a),^[17]^ which are much more reactive towards esterification[^18^, ^19^] or the formation of urethanes,^[20]^ thus opening new applications for lignins in polymer science.

In our previous study on soda lignin, we showed that a quantitative conversion of phenolic OH groups could be achieved in only 15 min at 150 °C.^[17]^ Recently, Liu et al. showed that softwood Kraft lignin could react efficiently with EC from 90 °C. They used 120 °C as preferred reaction temperature.^[18]^ A significant drawback observed during the modification of various lignins with EC is the formation of high molar mass fragments,^[18]^ which can even lead to complete insolubility in solvents.^[26]^ This is likely to be caused by crosslinking side reactions, which can occur by transcarbonation between a terminal primary OH group and a carbonate linkage (Scheme 1c). The latter are formed during the reaction of the aliphatic OH groups of lignin with EC (Scheme 1b). Crosslinking side reactions are much more important when lignin is modified with EC than with other cyclic carbonates, such as propylene, butylene or vinyl ethylene carbonates.[^16^, ^17^, ^26^] Indeed, the reaction with EC creates primary OH groups that are more prone to react by transcarbonation than the secondary OH groups which are mostly produced with substituted cyclic carbonates.^[17]^

Crosslinking seems to be favored by high reaction temperatures,[^18^, ^26^] which led us to look for milder reaction conditions for the modification of lignin with EC. Reactions were first performed with beech lignin at 90 °C, using 10 eq. EC and 0.1 eq K_2_CO_3_ as catalyst, as in our previous work.^[17]^ The reaction was followed by ^1^H NMR, which did not show a significant reduction in the signal of phenolic OH protons after 2 h (7.8–10 ppm, Supporting Information, Figure S14). At 100 °C, complete disappearance of phenolic OH was observed after 2 h by ^1^H NMR (Supporting Information, Figure S15) and confirmed by ^31^P NMR (Supporting Information, Figure S16). At 110 °C, more than 90 % of the phenolic OH were already converted in 30 min, and the conversion was total after 1 h, as seen on ^31^P NMR spectra (Supporting Information, Figure S17). The formation of carbonate linkages during the reaction of aliphatic OH groups is attested by the appearance of a shoulder at 1750 cm^−1^ on the corresponding FTIR spectra (Supporting Information, Figures S18 to S20).^[18]^

**Figure 8:**
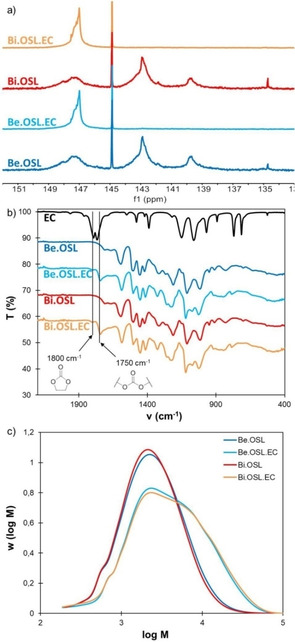
Characterization of beech and birch lignins before and after modification with EC (10 eq. EC, 0.1 eq K_2_CO_3_, 110 °C, 1 h): (a) ^31^P NMR spectra, (b) FTIR spectra, (c) SEC distributions.

Optimum reaction conditions (1 h at 110 °C) were then also applied to birch lignin, and the modified lignins were characterized by ^31^P NMR, FTIR and SEC. For both lignins, complete conversion of the phenolic OH groups is observed by ^31^P NMR (Figure 8a). The modified lignins thus only contain aliphatic OH groups, which contents increase by 75–85 % (Table 2). The signals of the grafted ethylene oxide units overlap with other lignin‐related signals in ^1^H NMR spectra (Supporting Information, Figure S15), thus preventing the determination of the average length of the grafts. The formation of carbonate linkages during the reaction of aliphatic OH groups with EC (Scheme 1b) is shown by the appearance of peaks at 1750 cm^−1^ on the FTIR spectra (Figure 8b). The molar mass increases after the reaction as a result of the grafting of ethylene oxide or ethylene carbonate groups, but the formation of high molar mass fragments is limited thanks to the mild conditions applied (Figure 8c). The weight‐average molar mass *M_w_* and the dispersity *Đ* increase, indicating that some crosslinking could have occurred, but in much lower proportion than in other studies employing harsher conditions.[^18^, ^19^, ^26^] As discussed earlier, crosslinking can occur *via* the formation of carbonate linkages and transcarbonation reactions (Scheme 1c), but other lignin condensation reactions can also occur. To evaluate them, a blank reaction was performed in which lignin was dissolved in a non‐reactive solvent (DMF) in the presence of catalyst and heated for 1 h at 110 °C. The results reported in the Supporting Information (Figure S21) show that this treatment causes a slight increase in molar mass, which is however much lower than during reaction with EC. It thus seems that the formation of high molar mass fragments is caused by the formation of carbonate linkages, although the occurrence of other lignin crosslinking reaction cannot be totally excluded.

**Table 2:** Properties of beech and birch lignins modified with EC.^[a]^

Sample name	EC [eq.]	Yield^[b]^ [%]	PhOH conv.^[c]^ [%]	Al−OH [mmol g^−1^]	Δ[Al−OH]^[d]^ [%]	*M*_n_ [g mol^−1^]	*M*_w_ [g mol^−1^]	Đ
Be.OSL	–	–	–	1.89	–	1810	3610	1.99
Be.OSL.EC	10	81±1	100	3.31±0.09	75±5	2730±30	7460±150	2.73±0.03
Bi.OSL	–	–	–	1.79	–	1760	3440	1.95
Bi.OSL.EC	10	87	100	3.34±0.20	86±11	2675±55	8320±390	3.11±0.21

[a] Conditions: 0.1 eq. K2 C03, 110 °C, 1 h; [b] Calculated according to Equation 10; [c] Calculated from ^31^P NMR results using Equation (8); [d] Calculated from ^31^P NMR results using Equation (9).

The reproducibility of the process was assessed by performing 5 replicates of the reaction with beech lignin and 3 with birch lignin. The results presented in the Supporting Information show an optimal reproducibility regarding the yields, conversion and molar masses (Figures S22 and S23 and Tables S6 and S7).

### Influence of lignin structure and molar mass on the reactivity with EC

To gain further insights into the reactivity of lignins, the fractions F1 to F5 isolated from beech lignin were also reacted with EC. Table 3 presents the results of the experiments and ^31^P NMR and FTIR spectra are available in the Supporting Information (Figures S24 and S25). The fractions were first reacted with the conditions successfully applied to the unfractionated lignins (10 eq. EC, 0.1 eq. K_2_CO_3_, 1 h at 110 °C). The lowest molar mass fractions F1 and F2 reacted easily, and full conversion of phenolic OH groups was achieved under these conditions. However, since the OH content of the fractions decreases from F1 to F5 (Figure 6), the use of a constant ratio of 10 eq. EC per OH groups leads to an increase of the lignin concentration in EC from 0.19 to 0.42 g mL^−1^ (Table 3). When the lignin concentration was higher than about 0.33 g mL^−1^, i. e. for fractions F3 to F5, the lignin could not be homogeneously dissolved in EC, ultimately leading to the recovery of an insoluble product that could not be analyzed.

**Table 3:** Properties of the beech lignin fractions modified with EC depending on the reaction conditions.^[a]^

Lignin fraction	EC [eq]	Lignin conc. [g mL^−1^]	*t* [h]	PhOH conv.^[b]^ [%]	AlOH [mmol g^−1^]	Δ(Al−OH)^[c]^ [%]	*M*_n_ [g mol^−1^]	*M*_w_ [g mol^−1^]	Đ
Be.OSL	10	0.27	1	100	3.31	75	2730	7460	2.73
F1	10	0.19	1	100	3.68	142	2180	5130	2.35
F2	10	0.31	1	100	3.40	123	–^[d]^	–^[d]^	–^[d]^
	12	0.25	1	100	3.35	120	2570	6530	2.54
F3	10	0.33	1	ins^[e]^	ins^[e]^	ins^[e]^	ins^[e]^	ins^[e]^	ins^[e]^
	13	0.25	1	100	3.08	71	3160	9230	2.92
	16	0.21	1	100	3.22	79	3090	8420	2.72
F4	10	0.35	1	ins^[e]^	ins^[e]^	ins^[e]^	ins^[e]^	ins^[e]^	ins^[e]^
	14	0.26	1	ins^[e]^	ins^[e]^	ins^[e]^	ins^[e]^	ins^[e]^	ins^[e]^
	18	0.20	1	100	3.31	108	3450	10510	3.05
F5	10	0.42	1	ins^[e]^	ins^[e]^	ins^[e]^	ins^[e]^	ins^[e]^	ins^[e]^
	21	0.26	1	0	1.76	44	–^[d]^	–^[d]^	–^[d]^
	21	0.26	2	ins^[e]^	ins^[e]^	ins^[e]^	ins^[e]^	ins^[e]^	ins^[e]^
	27.5	0.20	1	12	1.75	43	–^[d]^	–^[d]^	–^[d]^
	27.5	0.20	2	ins^[e]^	ins^[e]^	ins^[e]^	ins^[e]^	ins^[e]^	ins^[e]^
	10^[f]^	0.42^[f]^	1	100	2.10	72	ins^[e]^	ins^[e]^	ins^[e]^

[a] Conditions: 0.1 eq. K_2_CO_3_, 110 °C; [b] calculated from ^31^P NMR results using Equation (8); [d] calculated from ^31^P NMR results using Equation (9); [d] not measured; [e] ins=recovered product was insoluble; [f] DMF as solvent (2 mL per 250 mg lignin).

For these fractions, it was thus necessary to increase the amount of EC. Full conversion of the phenolic OH groups in F3 and F4 could be achieved with 16 and 18 eq., respectively. The high molar mass fraction F5 was more recalcitrant to functionalization. With 21 eq. EC, none of the phenolic OH groups reacted. Increasing the reaction time to 2 h led to the recovery of an insoluble product. With 27.5 eq. EC, only a moderate 12 % of conversion of the phenolic OH could be achieved and increasing the reaction time also led to crosslinked systems. This clearly evidences that the reactivity of lignin gets lower as the molar mass increases. Since the reaction is performed with EC as reactive solvent, it is possible that the solubility of the lignin in EC decreases when the molar mass increases, progressively leading to a decrease in reactivity. To test this phenomenon, a reaction was performed on F5 with 10 eq. EC using DMF as solvent. Full reactivity of the phenolic OH groups was observed after 1 h. This confirms that the observed decrease in reactivity is caused by the reduced solubility of the high molar mass lignin fragments in EC, rather than to a reduced accessibility or reactivity of the phenolic OH groups.

**Figure 9:**
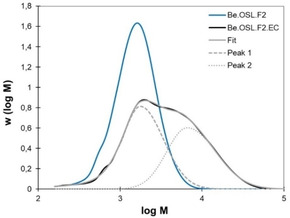
Molar mass distribution of fraction F2 from beech lignin before and after reaction with EC (10 eq. EC, 0.1 eq K_2_CO_3_, 110 °C, 1 h). The molar mass distribution of the modified lignin fraction was deconvoluted into two peaks.

The lignin fractions from the solvent fractionation have well‐defined, almost monodisperse molar mass distributions (Figure 5), which is highly valuable for an in‐depth study of the impact of the modification with EC. In all cases, the molar mass increases as a result of the grafting, and a significant increase in dispersity is observed (Table 3). Interestingly, the molar mass distributions of the modified lignin fractions could be deconvoluted, showing that they result from two distinct contributions. Figure 9 presents the results obtained for the fraction F2, and similar data for the other fractions are given in the Supporting Information (Figures S26). In all cases, the modified lignins seem to be composed of a low dispersity fraction, with a mass at the peak *M_p_* slightly higher than the unmodified lignin (peak 1 on Figure 9), and of a more disperse fraction with an *M_p_* about 3 to 4 times higher (peak 2 on Figure 9).

These results seem to support the proposed reaction mechanisms in Scheme 1, indicating that two different reaction pathways occur during the reaction of lignin with EC. The lignin reacts according to the pathway described on Scheme 1a and b, which leads to a moderate increase in the apparent molar mass, because the grafted chemical groups are relatively small and do not lead to a marked increase in the hydrodynamic radius. Concomitantly, some chain coupling seems to occur, probably by transcarbonation, as depicted on Scheme 1c, leading to the formation of fragments of higher molar mass and dispersity. This side reaction has two major drawbacks: it consumes some of the newly formed aliphatic OH groups, leading to a decrease in the OH content of the modified lignin, and it increases the molar mass rather quickly. If not well controlled, this can even lead to the formation of an insoluble network, as pointed out in other studies.^[26]^ These results thus show the necessity of a precise control of the reaction conditions, as developed here, in order to minimize the crosslinking and maximize the potential of the lignins modified with EC.

## Conclusion

Organosolv lignins from beech and birch isolated during scale‐up runs of the acetone‐based Fabiola™ process were first characterized. Their solubility in various common organic solvents was evaluated and analyzed with respect to Hansen and Kamlet‐Taft solubility parameters. The solubility was found to be well correlated to the Kamlet‐Taft dipolarity/polarizability π* parameter, which could be meaningful to predict the solubility of lignins in organic solvents. The possibility to extend this correlation to other kinds of technical lignins should now be evaluated.

The results of the solubility tests were then exploited to select a set of solvents able to isolate lignin fractions in a sequential solvent fractionation. Five fractions were recovered in equilibrated yields (15 to 25 wt%), with similar results for both lignin sources. They show low dispersity with a phenolic OH groups content that is inversely correlated to the average molar mass.

Since we have shown in previous studies that the modification of lignins with cyclic carbonates is a robust and green approach to develop highly valuable aromatic building blocks for macromolecular architectures,[^15^, ^16^, ^17^] both lignins were then modified with ethylene carbonate (EC), to convert the phenolic OH into more reactive primary aliphatic OH groups. The reaction leads to complete modification of the phenolic OH groups for both lignins with milder reaction conditions than previously reported (1 h at 110 °C), highlighting the high reactivity of the studied acetone organosolv lignins. The study of the reactivity of the fractions isolated via sequential solvent fractionation leads to interesting observations. The reactivity decreases gradually when the molar mass increases, and the fraction of highest molar mass is almost unreactive under the studied conditions. This was shown to result from the decreasing solubility of lignin in EC as the molar mass increases. Detailed study of the molar mass distributions of the modified lignin fractions reveals that two distinct reactions may occur during lignin modification with EC: the grafting of ethylene oxide or carbonate units, and the crosslinking, probably *via* transcarbonation reactions. The latter presents significant drawbacks as it reduces the content in OH groups and quickly increases the molar mass of the modified lignins, but our results show that it can be minimized by choosing optimized reaction conditions. The use of the modified lignins as building blocks for the synthesis of different kind of polymers, such as polyesters and polyurethanes, is currently under development in our laboratory.

## Experimental Section

### Materials

Lignins from beech (Be.OSL) and birch (Bi.OSL) were isolated from pilot runs (460 L scale) of the acetone organosolv Fabiola™ process^[21]^ performed at Fraunhofer CBP (Leuna, Germany). After fractionation, the lignin was precipitated from the liquor by dilution with three volumes of water and recovered by filtration using a chamber filter press. The lignins were dried overnight in a vacuum oven at 40 °C before analysis.

Ethyl acetate (EtOAc, ≥99.8 %), methanol (MeOH, ≥99.9 %), pyridine (≥99.9 %) and dimethylformamide (DMF, synthesis grade) were purchased from Fisher Scientific, ethanol (EtOH, absolute, ≥99.5 %), isopropanol (*i*‐prOH, GPR Rectapur) and acetone (technical grade) from VWR, methyl ethyl ketone (MEK, 2‐butanone, technical grade) and ethylene carbonate (EC, ≥99 %) from Acros Organics, 1‐propanol (1‐prOH, ≥99.9 %) from Alfa Aesar and dimethylsulfoxide (DMSO, anhydrous, ≥99.9 %) from Sigma‐Aldrich. The chemicals were all used as received without purification.

### Lignins solubility tests in organic solvents

Dry lignin (2 g) were suspended in 20 mL solvent at room temperature, stirred for 2 h, and filtered over 0.45 μm PVDF membranes (Durapore®, Merck Millipore). The insoluble part was then re‐suspended in 20 mL fresh solvent for 2 h, and the operation was repeated. The insoluble fraction was then dried in a vacuum oven at 40 °C overnight, and its final mass *m*_ins_ was measured. The soluble fractions were combined and evaporated to dryness in a rotary evaporator, followed by additional drying in a vacuum oven at 40 °C overnight, and finally weighed (*m*_sol_).

The soluble and insoluble weight fractions were then calculated as follows [Eq. (6) and 7]:

(6) $$Soluble\;fraction\;\left(\%wt\right)=\;\frac{m_{sol}}{m_{i}}\;\times 100$$

(7) $$Insoluble\;fraction\;\left(\%wt\right)=\;\frac{m_{ins}}{m_{i}}\;\times 100$$

where *m*_i_ is the initial mass of lignin (2 g).

The tested solvents were ethyl acetate (EtOAc), methanol (MeOH), ethanol (EtOH), 1‐propanol (1‐PrOH), isopropanol (i‐PrOH), acetone and methyl ethyl ketone (MEK).

### Sequential solvent fractionation

Based on the solubility tests, 4 solvents were selected for the sequential solvent fractionation and used in the following order: (i) 1‐propanol, (ii) ethanol, (iii) methanol and (iv) acetone. 5 g of dry lignin (Be.OSL or Bi.OSL) were suspended in 50 mL of the first solvent and treated as described for the solubility tests. At the end of each step, the insoluble fraction was suspended in the following solvent and treated as before. Yields were calculated based on the initial lignin weight. To evaluate the scalability, the sequential solvent fractionation was also reproduced with 50 g of Be.OSL. The amount of solvent was also scaled by a factor 10 to ensure similar concentrations as in the small‐scale fractionation.

### Chemical modification of lignin with Ethylene Carbonate (EC)

The reaction with ethylene carbonate (EC) was performed following the protocol previously described with slight modifications.^[17]^ Lignin (0.25–10 g), K_2_CO_3_ (0.1 molar equivalent with respect to reactive groups, i. e. the sum of OH and COOH groups) and EC (10 molar equivalents) were added to a round‐bottom flask, which was flushed with argon and immersed in an oil bath regulated at 110 °C. After 1 h of reaction, the reaction mixture was poured into cold water previously acidified to pH 2 by the addition of a 2 m HCl solution, leading to the precipitation of the modified lignin. It was recovered by filtration, washed on the filter with acidified water, and dried overnight in a vacuum oven at 40 °C.

The modified lignins were then characterized by ^31^P NMR. Their content in aliphatic and phenolic OH groups, Al−OH and Ph−OH, were compared to the same in the unmodified lignin, Al−OH_0_ and Ph−OH_0_. The conversion of the phenolic OH groups was determined using Equation 8:

(8) $$Ph-OH\;conversion\;\left(\%\right)=\;\frac{Ph-OH}{{Ph-OH}_{0}}\;\times 100$$

The increase in aliphatic OH groups, Δ(Al−OH), was calculated using Equation 9:

(9) $$\Delta \left(Al-OH\right)=\;\frac{Al-OH-\;{Al-OH}_{0}}{{Al-OH}_{0}}\;\times 100$$

The yield was calculated taking into account the increase in mass due to the grafting, as previously reported.[^16^, ^63^] The grafting of ethylene oxide groups onto the phenolic OH groups leads to an increase of 44 g mol^−1^, whereas the grafting of ethylene carbonate groups onto the aliphatic OH leads to an increase of 88 g mol^−1^. The yield was thus calculated according to Equation 10:

(10) $$Yield\;\left(\%\right)=\;\frac{m_{f}}{m_{i}\;\left(1+\left[Ph-OH\right]\;\times 44+\left[Al-OH\right]\times 88\right)}$$

where *m*_i_ and *m*_f_ are the initial and final masses, [Ph−OH] and [Al−OH] are the content in phenolic and aliphatic OH of the starting lignin (in mol g^−1^).

### Blank reaction

To evaluate the impact of the temperature and catalyst on the lignin, a blank reaction was performed in a non‐reactive solvent. 250 mg lignin were dissolved in 1 mL dry DMF in a round bottom flask, and 19 mg K_2_CO_3_ (0.1 molar equivalent with respect to lignin reactive groups, i. e. the sum of OH and COOH groups) were added. The flask was equipped with a reflux condenser, flushed with argon, and immersed in an oil bath regulated at 110 °C for 1 h. The reaction mixture was then poured into cold water previously acidified to pH 2 by the addition of a 2 m HCl solution, leading to the precipitation of the lignin, which was recovered by filtration, washed on the filter with acidified water, and dried overnight in a vacuum oven at 40 °C. 231 mg lignin (92 % yield) were recovered.

### Lignin characterization

The total lignin content was determined according to the simplified protocol described by Aldaeus et al.^[48]^ About 100 mg samples were accurately weighed in a beaker, followed by the addition of 1 mL 72 wt% H_2_SO_4_ solution and 28 mL water. The suspensions were then heated to 80 °C and filtered while still hot. The acid‐insoluble lignin was measured gravimetrically, according to TAPPI T 222 om‐02 method. The acid‐soluble lignin was measured on the filtrates by UV spectroscopy on a Shimadzu UV 2600 spectrometer, assuming an extinction coefficient of 110 L g^−1^ cm^−1^ at 205 nm wavelength, according to TAPPI UM 250 method.

Size Exclusion Chromatography (SEC) was performed on a Waters Acquity Advanced Polymer Chromatography (APC) system, equipped with three 150 mm APC XT columns (a 45 Å, 1.7 μm, a 200 Å, 2.5 μm and a 450 Å, 2.5 μm) thermostated at 40 °C. Tetrahydrofuran (THF, HPLC grade, Fisher Scientific) was used as eluent at a flow rate of 0.6 mL min^−1^. The detection was performed by an Acquity refractive index (RI) detector and an Acquity TUV detector operating at 280 nm. To ensure full solubility in THF, all samples were first acetylated according to a standard protocol,^[63]^ then dissolved in THF at 5 mg mL^−1^ concentration and filtered through 0.2 μm PTFE syringe filters prior to injection. The average molar masses (*M*_n_, *M*_w_) and dispersities (*Đ*) were calculated from a calibration with polystyrene standards.

NMR spectroscopy was performed on a Bruker 400 MHz spectrometer. ^31^P NMR was measured after derivatization of the samples with 2‐chloro‐4,4,5,5‐tetramethyl‐1,3,2‐dioxaphospholane (95 %, Sigma‐Aldrich) in pyridine/CDCl_3_ (1.6 : 1 *v*/*v*), in the presence of cholesterol as internal standard, according to the standard protocol.[^64^, ^65^] 128 scans were collected at 25 °C with a 15 s delay. For ^1^H NMR, the samples were dissolved in 550 μL DMSO‐*d*_6_ and 100 μL of a standard solution of 2,3,4,5,6‐pentafluorobenzaldehyde (0.5 m in DMSO‐*d*_6_) was added. 32 scans were recorded at 25 °C with a 15 s delay.

FTIR spectroscopy was performed on a Nicolet 380 spectrometer in attenuated total reflectance (ATR) mode. Lignin samples in powder form were directly deposited on the ATR crystal, and 32 scans were collected between 500 and 4000 cm^−1^ at 4 cm^−1^ resolution.

Differential scanning calorimetry (DSC) was performed on a TA Q200 calorimeter. The samples (2–3 mg) were first heated at 10 °C min^−1^ to 105 °C and maintained at this temperature for 15 min to erase the thermal history. They were then cooled to 0 °C at 10 °C min^−1^, maintained at 0 °C for 3 min and heated to 200 °C at 10 °C min^−1^. The glass transition temperature (*T*_g_) was taken as the midpoint of the change in slope during the second heating run.

## Conflict of interest

The authors declare no conflict of interest.

## Supporting information

As a service to our authors and readers, this journal provides supporting information supplied by the authors. Such materials are peer reviewed and may be re‐organized for online delivery, but are not copy‐edited or typeset. Technical support issues arising from supporting information (other than missing files) should be addressed to the authors.

Supplementary

## References

[1] Z. Zhou , F. Lei , P. Li , J. Jiang , Biotechnol. Bioeng. 2018, 115, 2683–2702. 29959859 10.1002/bit.26788

[2] M. N. Borand , F. Karaosmanoğlu , J. Renewable Sustainable Energy 2018, 10, 033104.

[3] M. Galbe , O. Wallberg , Biotechnol. Biofuels 2019, 12, 294. 31890022 10.1186/s13068-019-1634-1 PMC6927169

[4] P. P. Thoresen , L. Matsakas , U. Rova , P. Christakopoulos , Bioresour. Technol. 2020, 306, 123189. 32220471 10.1016/j.biortech.2020.123189

[5] Z. Jiang , P. Zhao , C. Hu , Bioresour. Technol. 2018, 256, 466–477. 29478782 10.1016/j.biortech.2018.02.061

[6] S. S. Hassan , G. A. Williams , A. K. Jaiswal , Trends Biotechnol. 2019, 37, 231–234. 30049417 10.1016/j.tibtech.2018.07.002

[7] A. Duval , M. Lawoko , React. Funct. Polym. 2014, 85, 78–96.

[8] S. Laurichesse , L. Avérous , Prog. Polym. Sci. 2014, 39, 1266–1290.

[9] B. M. Upton , A. M. Kasko , Chem. Rev. 2016, 116, 2275–2306. 26654678 10.1021/acs.chemrev.5b00345

[10] W. G. Glasser , C. A. Barnett , T. G. Rials , V. P. Saraf , J. Appl. Polym. Sci. 1984, 29, 1815–1830.

[11] L. C. F. Wu , W. G. Glasser , J. Appl. Polym. Sci. 1984, 29, 1111–1123.

[12] C. A. Cateto , M. F. Barreiro , A. E. Rodrigues , M. N. Belgacem , Ind. Eng. Chem. Res. 2009, 48, 2583–2589.

[13] H. Sadeghifar , C. Cui , D. S. Argyropoulos , Ind. Eng. Chem. Res. 2012, 51, 16713–16720.

[14] S. Laurichesse , C. Huillet , L. Avérous , Green Chem. 2014, 16, 3958–3970.

[15] A. Duval , L. Avérous , ACS Sustainable Chem. Eng. 2016, 4, 3103–3112.

[16] A. Duval , L. Avérous , ChemSusChem 2017, 10, 1813–1822. 28195674 10.1002/cssc.201700066

[17] A. Duval , L. Avérous , ACS Sustainable Chem. Eng. 2017, 5, 7334–7343.

[18] L.-Y. Liu , M. Cho , N. Sathitsuksanoh , S. Chowdhury , S. Renneckar , ACS Sustainable Chem. Eng. 2018, 6, 12251–12260.

[19] L.-Y. Liu , Q. Hua , S. Renneckar , Green Chem. 2019, 21, 3682–3692.

[20] X. Zhang , Y. Kim , I. Elsayed , M. Taylor , T. L. Eberhardt , E. B. Hassan , R. Shmulsky , Ind. Crops Prod. 2019, 141, 111797.

[21] A. Smit , W. Huijgen , Green Chem. 2017, 19, 5505–5514.

[22] A. Duval , F. Vilaplana , C. Crestini , M. Lawoko , Holzforschung 2016, 70, 11–20.

[23] H. Sadeghifar , A. Ragauskas , ACS Sustainable Chem. Eng. 2020, 8, 8086–8101.

[24] M. Gigli , C. Crestini , Green Chem. 2020, 22, 4722–4746.

[25] J. Xu , C. Li , L. Dai , C. Xu , Y. Zhong , F. Yu , C. Si , ChemSusChem 2020, 13, 4284–4295. 32672385 10.1002/cssc.202001491

[26] I. Kühnel , B. Saake , R. Lehnen , React. Funct. Polym. 2017, 120, 83–91.

[27] K. Alfonsi , J. Colberg , P. J. Dunn , T. Fevig , S. Jennings , T. A. Johnson , H. P. Kleine , C. Knight , M. A. Nagy , D. A. Perry , M. Stefaniak , Green Chem. 2008, 10, 31–36.

[28] R. K. Henderson , C. Jiménez-González , D. J. C. Constable , S. R. Alston , G. G. A. Inglis , G. Fisher , J. Sherwood , S. P. Binks , A. D. Curzons , Green Chem. 2011, 13, 854–862.

[29] D. Prat , O. Pardigon , H.-W. Flemming , S. Letestu , V. Ducandas , P. Isnard , E. Guntrum , T. Senac , S. Ruisseau , P. Cruciani , P. Hosek , Org. Process Res. Dev. 2013, 17, 1517–1525.

[30] D. Prat , J. Hayler , A. Wells , Green Chem. 2014, 16, 4546–4551.

[31] D. Prat , A. Wells , J. Hayler , H. Sneddon , C. R. McElroy , S. Abou-Shehada , P. J. Dunn , Green Chem. 2016, 18, 288–296.

[32] R. Mörck , H. Yoshida , K. P. Kringstad , Holzforschung 1986, 40, 51–60.

[33] R. J. A. Gosselink , J. E. G. van Dam , E. de Jong , E. L. Scott , J. P. M. Sanders , J. Li , G. Gellerstedt , Holzforschung 2010, 64, 193–200.

[34] A. P. Dodd , J. F. Kadla , S. K. Straus , ACS Sustainable Chem. Eng. 2015, 3, 103–110.

[35] M. N. Vanderlaan , R. W. Thring , Biomass Bioenergy 1998, 14, 525–531.

[36] J. Ropponen , L. Rasanen , S. Rovio , T. Ohra-aho , T. Liitia , H. Mikkonen , D. van de Pas , T. Tamminen , Holzforschung 2011, 65, 543–549.

[37] J. R. D. Montgomery , C. S. Lancefield , D. M. Miles-Barrett , K. Ackermann , B. E. Bode , N. J. Westwood , T. Lebl , ACS Omega 2017, 2, 8466–8474. 31457383 10.1021/acsomega.7b01287 PMC6645228

[38] J. H. Hildebrand , R. L. Scott , The Solubility of Nonelectrolytes, Reinhold Pub. Corp., New York, 1950.

[39] C. M. Hansen , Hansen Solubility Parameters: A User's Handbook, CRC Press, Boca Raton, FL, 2007.

[40] C. Schuerch , J. Am. Chem. Soc. 1952, 74, 5061–5067.

[41] N. Giummarella , C. Lindgren , M. E. Lindström , G. Henriksson , BioResources 2016, 11, 3494–3510.

[42] C. M. Hansen , A. Björkman , Holzforschung 1998, 52, 335–344.

[43] G. Cañete Vebber , P. Pranke , C. Nunes Pereira , J. Appl. Polym. Sci. 2014, 131, 39696.

[44] L. P. Novo , A. A. S. Curvelo , Ind. Eng. Chem. Res. 2019, 58, 14520–14527.

[45] M. J. Kamlet , J. L. M. Abboud , M. H. Abraham , R. W. Taft , J. Org. Chem. 1983, 48, 2877–2887.

[46] R. W. Taft , J.-L. M. Abboud , M. J. Kamlet , M. H. Abraham , J. Solution Chem. 1985, 14, 153–186.

[47] A. Tagami , C. Gioia , M. Lauberts , T. Budnyak , R. Moriana , M. E. Lindström , O. Sevastyanova , Ind. Crops Prod. 2019, 129, 123–134.

[48] F. Aldaeus , H. Schweinebarth , P. Törngren , A. Jacobs , Holzforschung 2011, 65, 601–604.

[49] J. Ponomarenko , T. Dizhbite , M. Lauberts , A. Viksna , G. Dobele , O. Bikovens , G. Telysheva , BioResources 2014, 9, 2051–2068.

[50] D. Barana , M. Orlandi , L. Zoia , L. Castellani , T. Hanel , C. Bolck , R. Gosselink , ACS Sustainable Chem. Eng. 2018, 6, 11843–11852.

[51] C. Gioia , G. Lo Re , M. Lawoko , L. Berglund , J. Am. Chem. Soc. 2018, 140, 4054–4061. 29498848 10.1021/jacs.7b13620

[52] S. Gouveia , C. Fernández-Costas , M. A. Sanromán , D. Moldes , Bioresour. Technol. 2012, 121, 131–138. 22858477 10.1016/j.biortech.2012.05.144

[53] J.-Y. Kim , S. Y. Park , J. H. Lee , I.-G. Choi , J. W. Choi , RSC Adv. 2017, 7, 53117–53125.

[54] A. Majira , B. Godon , L. Foulon , J. C. van der Putten , L. Cézard , M. Thierry , F. Pion , A. Bado-Nilles , P. Pandard , T. Jayabalan , V. Aguié-Béghin , P.-H. Ducrot , C. Lapierre , G. Marlair , R. J. A. Gosselink , S. Baumberger , B. Cottyn , ChemSusChem 2019, 12, 4799–4809. 31436856 10.1002/cssc.201901916 PMC6899661

[55] S. Y. Park , J.-Y. Kim , H. J. Youn , J. W. Choi , Int. J. Biol. Macromol. 2019, 138, 1029–1034. 31356954 10.1016/j.ijbiomac.2019.07.157

[56] S. Y. Park , J.-Y. Kim , H. J. Youn , J. W. Choi , Int. J. Biol. Macromol. 2018, 106, 793–802. 28818728 10.1016/j.ijbiomac.2017.08.069

[57] V. Rohde , S. Böringer , B. Tübke , C. Adam , N. Dahmen , D. Schmiedl , GCB Bioenergy 2019, 11, 206–217.

[58] T. G. Fox , P. J. Flory , J. Appl. Phys. 1950, 21, 581–591.

[59] Z. Dobkowski , Eur. Polym. J. 1982, 18, 563–567.

[60] M. Jayakannan , S. Ramakrishnan , J. Polym. Sci. Part A 2000, 38, 261–268.

[61] X. Luo , S. Xie , J. Liu , H. Hu , J. Jiang , W. Huang , H. Gao , D. Zhou , Z. Lü , D. Yan , Polym. Chem. 2014, 5, 1305–1312.

[62] Y. H. Kim , R. Beckerbauer , Macromolecules 1994, 27, 1968–1971.

[63] A. Duval , L. Avérous , Green Chem. 2020, 22, 1671–1680.

[64] A. Granata , D. S. Argyropoulos , J. Agric. Food Chem. 1995, 43, 1538–1544.

[65] X. Meng , C. Crestini , H. Ben , N. Hao , Y. Pu , A. J. Ragauskas , D. S. Argyropoulos , Nat. Protoc. 2019, 14, 2627–2647. 31391578 10.1038/s41596-019-0191-1

