# Supplementary material for: Isolation of Low Dispersity Fractions of Acetone Organosolv Lignins to Understand their Reactivity: Towards Aromatic Building Blocks for Polymers Synthesis
Source: ChemSusChem. 2020 Oct 16;14(1):387–97. doi: 10.1002/cssc.202001976 (PMC7821138; doi:10.1002/cssc.202001976)
Supplement: Supplementary file 1 — Supplementary [file CSSC-14-387-s001.pdf]

ChemSusChem

Supporting Information

Isolation of Low Dispersity Fractions of Acetone Organosolv Lignins to Understand their Reactivity: Towards Aromatic Building Blocks for Polymers Synthesis

Antoine Duval,* Géraldine Layrac, André van Zomeren, Arjan T. Smit, Eric Pollet, and Luc Avérous*© 2020 The Authors. ChemSusChem published by Wiley-VCH GmbH. This is an open access article under the terms of the Creative Commons Attribution License, which permits use, distribution and reproduction in any medium, provided the original work is properly cited.

Solubility of Fabiola™ lignins in organic solvents

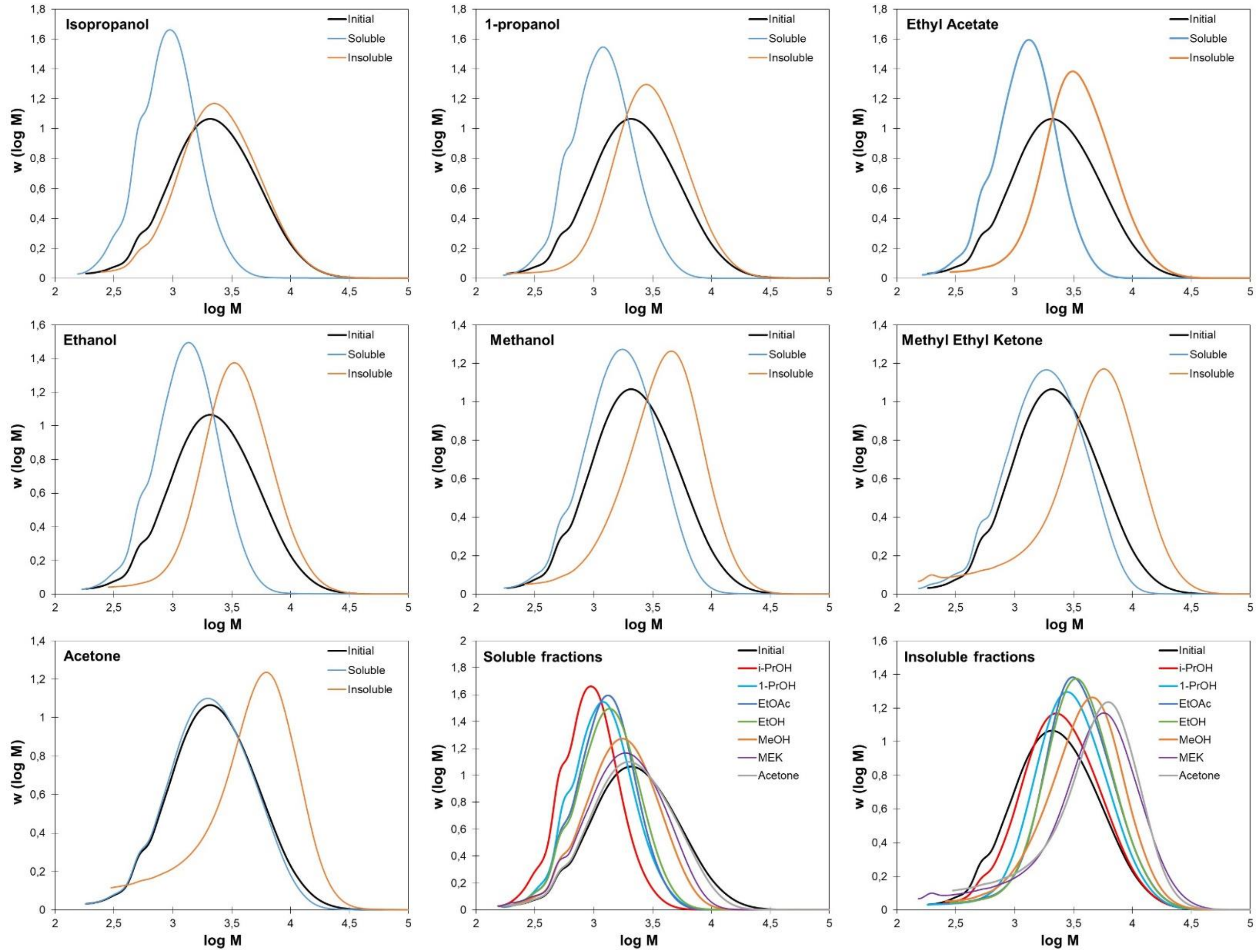

Figure S 1. SEC distributions of the soluble and insoluble fractions in various solvents of beech lignin (Be.OSL)

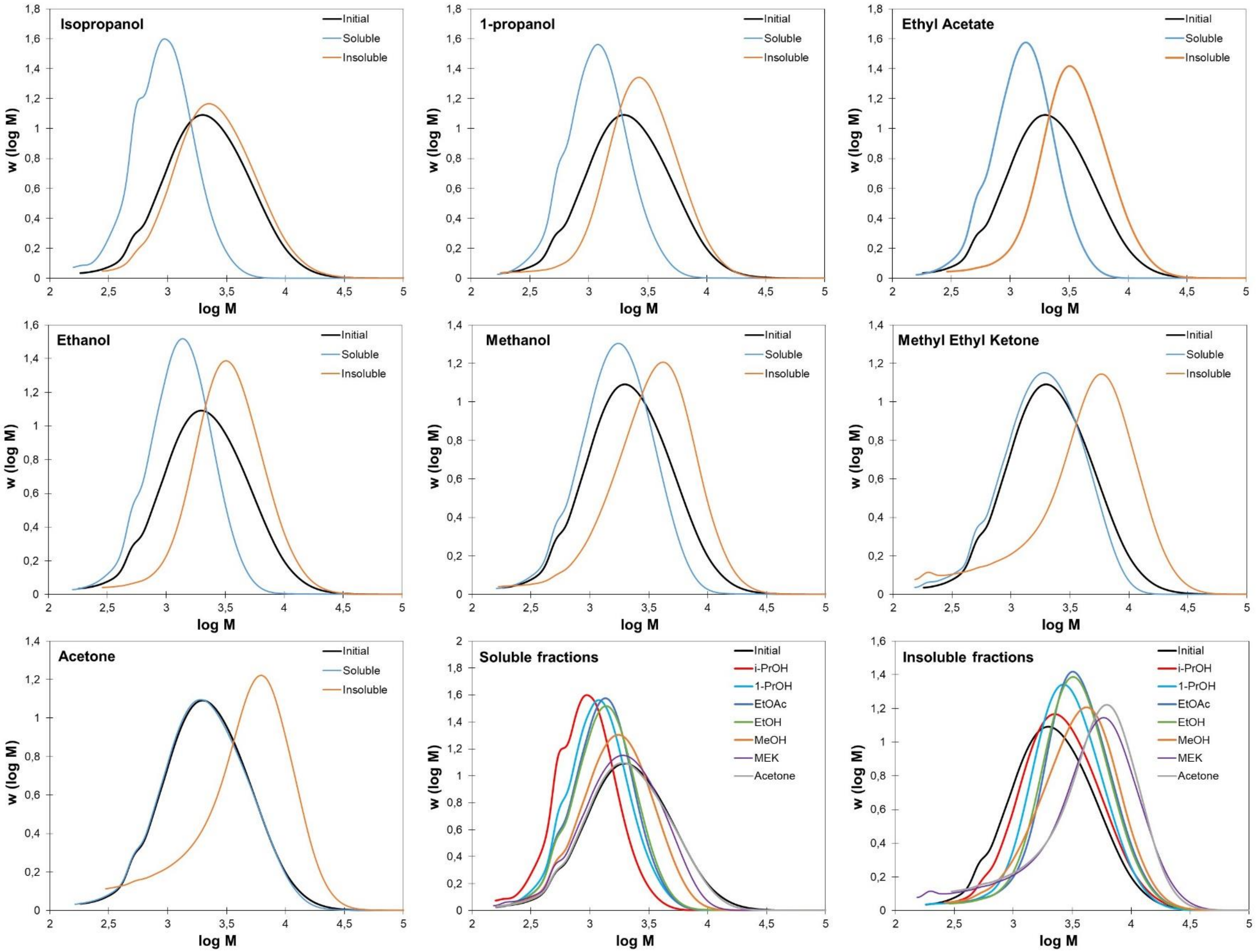

Figure S 2. SEC distributions of the soluble and insoluble fractions in various solvents of birch lignin (Bi.OSL)

Table S 1. Hansen[1] and Kamlet-Taft parameters[2,3] of the various solvents used in this study

Solvent	Hansen solubility parameters				Kamlet-Taft parameters		
	δ_D ($MPa^{1/2}$)	δ_P ($MPa^{1/2}$)	δ_H ($MPa^{1/2}$)	δ ($MPa^{1/2}$)	α	β	π^*
Methanol	15.1	12.3	22.3	29.6	0.98	0.66	0.60
Ethanol	15.8	8.8	19.4	26.5	0.86	0.75	0.54
1-propanol	16.0	6.8	17.4	24.6	0.84	0.90	0.52
Isopropanol	15.8	6.1	16.4	23.6	0.76	0.84	0.48
Acetone	15.5	10.4	7.0	19.9	0.08	0.43	0.71
MEK	16,0	9,0	5,1	19,1	0,06	0,48	0,67
Ethyl Acetate	15.8	5.3	7.2	18.2	0.00	0.45	0.55
DMSO	18.4	16.4	10.2	26.7	0.00	0.76	1.00
DMF	17.4	13.7	11.3	24.9	0.00	0.69	0.88
Pyridine	19.0	8.8	5.9	21.8	0.00	0.64	0.87

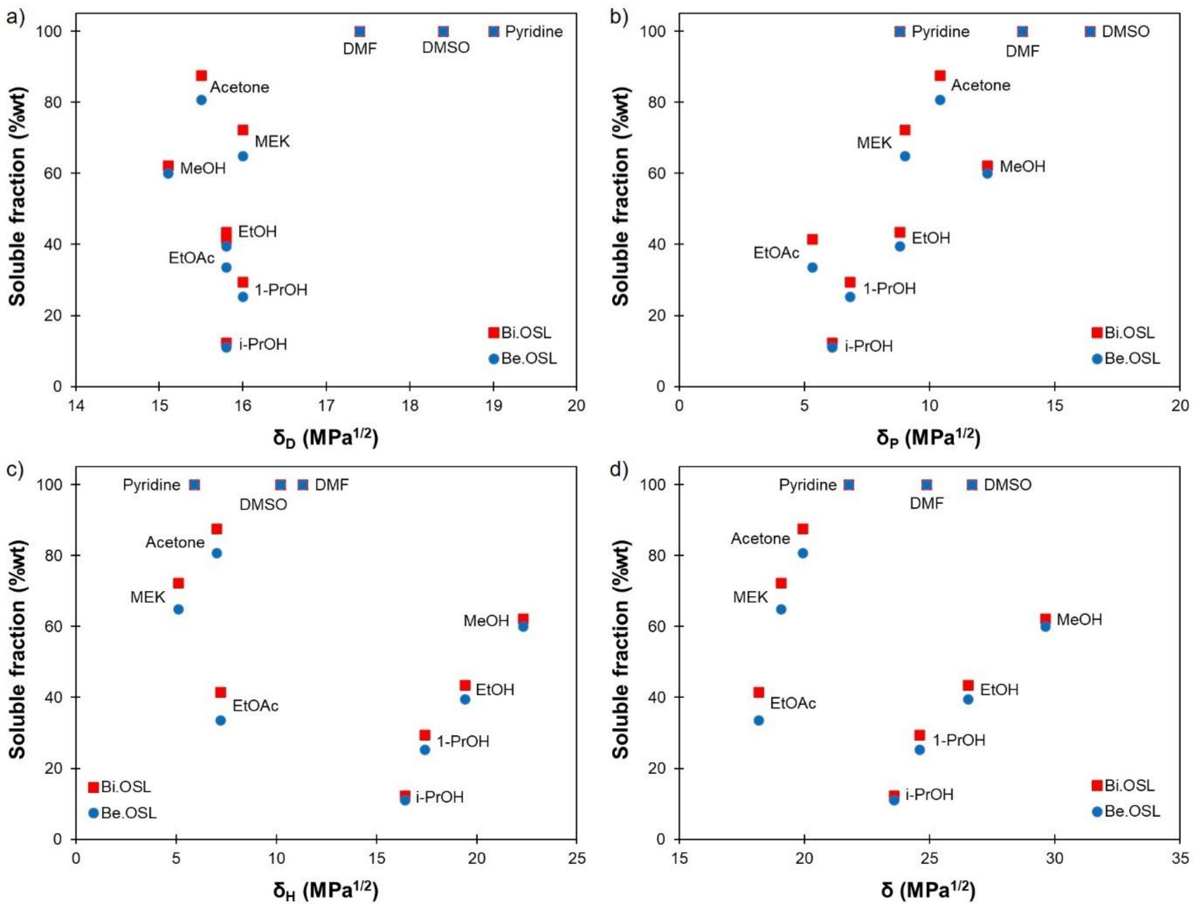

Figure S 3. Solubility of beech and birch lignins in various organic solvents depending on their Hansen solubility parameters: (a) dispersion δ_P, (b) polarity δ_H, (c) hydrogen-bonding ability δ_H and (d) Hildebrand solubility parameter δ

The Relative Energy Differences (RED) between lignin and the different solvents were calculated as:

$$RED = \frac{R_a}{R_0} \quad \text{(S1)}$$

with
$$R_a = \sqrt{4\,(\delta_{D1} - \delta_{D2})^2 + (\delta_{P1} - \delta_{P2})^2 + (\delta_{H1} - \delta_{H2})^2} \quad \text{(S2)}$$

Two sets of Hansen solubility parameters for lignin were tested (Table S 2).

Table S 2. Hansen Solubility Parameters (HSPs) of lignin according to different references

δ_D (MPa$^{1/2}$)	δ_P (MPa$^{1/2}$)	δ_H (MPa$^{1/2}$)	R_0 (MPa$^{1/2}$)	Reference
21.9	14.1	16.9	13.7	Hansen & Björkman[4]
21.71	14.19	16.93	13.45	Vebber et al.[5]
21.42	8.57	21.80	13.56	Novo & Curvelo[6]

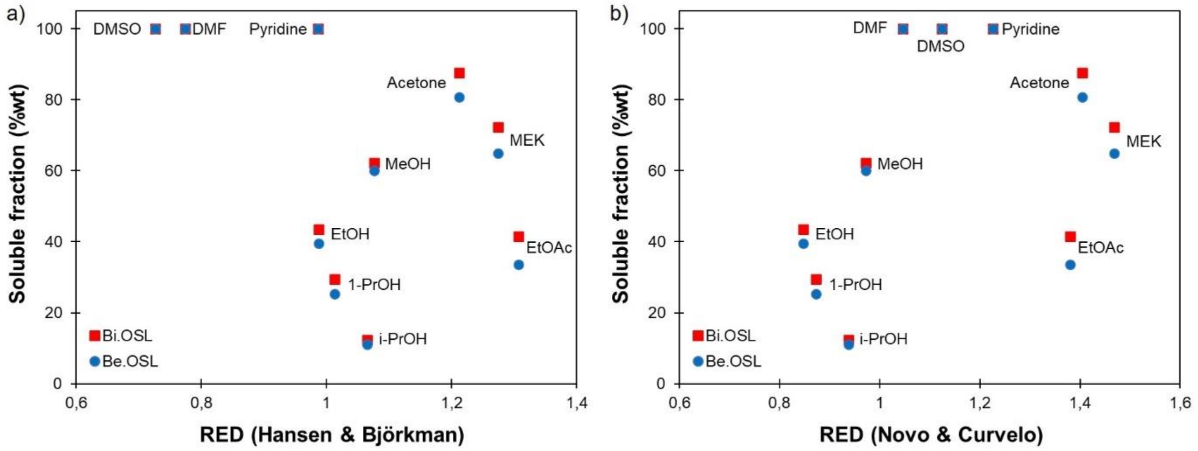

Figure S 4. Solubility of beech and birch lignins in various organic solvents depending on the Relative Energy Difference (RED, equation S1 – S2). Calculation was done with HSPs of lignin taken from: (a) Hansen & Björkman[4] and (b) Novo & Curvelo[6]

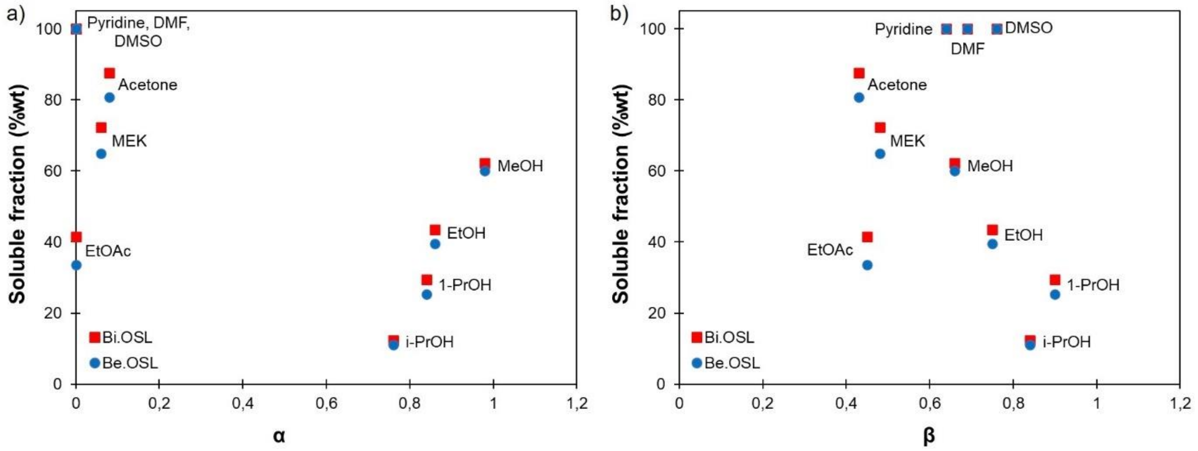

Figure S 5. Solubility of beech and birch lignins in various organic solvents depending on their Kamlet-Taft parameters: (a) hydrogen bond donor (HBD) ability α, (b) hydrogen bond acceptor (HBA) ability β

Sequential solvent fractionation: towards low dispersity lignin fractions

The yields of the sequential solvent fractionation were predicted from the results of the solubility tests, using the following equations, as already described (Equations S1 – S5):[7]

$$x_{F1} = x_{1-propanol} \quad (S1)$$

$$x_{F2} = x_{Ethanol} - x_{1-propanol} \quad (S2)$$

$$x_{F3} = x_{Methanol} - x_{Ethanol} \quad (S3)$$

$$x_{F4} = x_{Acetone} - x_{Methanol} \quad (S4)$$

$$x_{F5} = 100 - x_{Acetone} \quad (S5)$$

where *x* represents the soluble fraction in the corresponding solvent (see Table 1 in the manuscript).

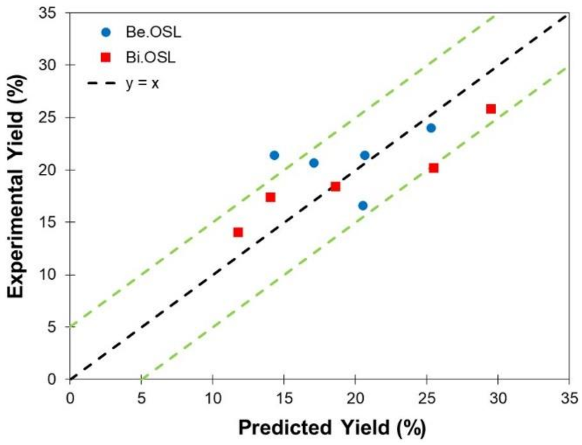

Figure S 6. Experimental yields of the solvent fractionation of beech and birch lignins as a function of the predicted yields. Green dotted lines correspond to y = x ± 5%

Table S 3. Properties of the fractions obtained by sequential solvent fractionation of beech and birch lignins

Lignin	Fraction	Yield (%wt)	M_n (g mol^{-1})	M_w (g mol^{-1})	Đ	Al-OH (mmol g^{-1})	Ph-OH (mmol g^{-1})	COOH (mmol g^{-1})
Be.OSL	- [a]	-	1810	3610	1.99	1.89	3.58	0.06
	F1	24.0	1020	1470	1.44	1.52	5.89	0.31
	F2	21.4	1500	2070	1.38	1.84	3.19	*nd* [b]
	F3	16.6	2410	3360	1.39	1.75	2.65	*nd* [b]
	F4	21.4	3070	4650	1.51	1.61	2.57	*nd* [b]
	F5	20.7	4270	8460	1.98	1.64	1.94	*nd* [b]
Bi.OSL	- [a]	-	1760	3440	1.95	1.79	3.76	0.09
	F1	25.8	1020	1470	1.44	1.03	5.18	0.32
	F2	17.4	1530	1990	1.30	1.46	3.21	*nd* [b]
	F3	18.4	2400	3260	1.36	1.65	2.56	*nd* [b]
	F4	20.2	3260	4940	1.52	1.57	2.33	*nd* [b]
	F5	14.0	3880	8340	2.15	1.62	1.88	0.07

[a] Initial lignin (not fractionated)

[b] *nd* = not detected

Table S 4. Acid soluble and insoluble lignin content of the fractions obtained by sequential solvent fractionation of beech and birch lignins

Lignin	Fraction	Acid insoluble lignin (%wt)	Acid soluble lignin (%wt)	Total lignin (%wt)
Be.OSL	- [a]	94.4	2.0	96.4
	F1	79.5	4.2	83.7
	F2	90.2	1.0	91.2
	F3	96.8	0.4	97.2
	F4	95.7	0.2	95.9
	F5	95.8	0.5	96.2
Bi.OSL	- [a]	92.0	1.7	93.7
	F1	80.7	3.5	84.1
	F2	92.4	0.8	93.2
	F3	95.9	0.4	96.3
	F4	94.1	0.2	94.4
	F5	95.7	0.9	96.7

[a] Initial lignin (not fractionated)

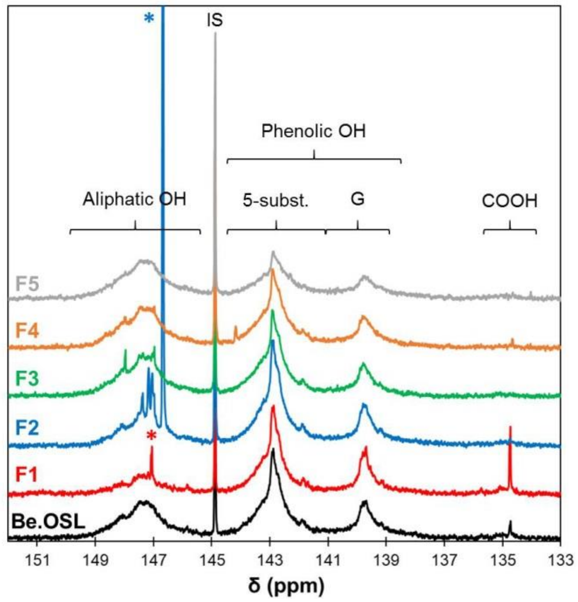

Figure S 7. ^{31}P NMR spectra of beech lignin and its fractions. Integration regions of the different functional groups were taken as described by Meng et al.[8] Peaks labeled with stars are residual peaks from solvents (1-propanol in F1 and ethanol in F2, respectively). Their integrals were deduced from the integrals of the aliphatic OH region to avoid an overestimation of lignin fractions content in aliphatic OH groups.

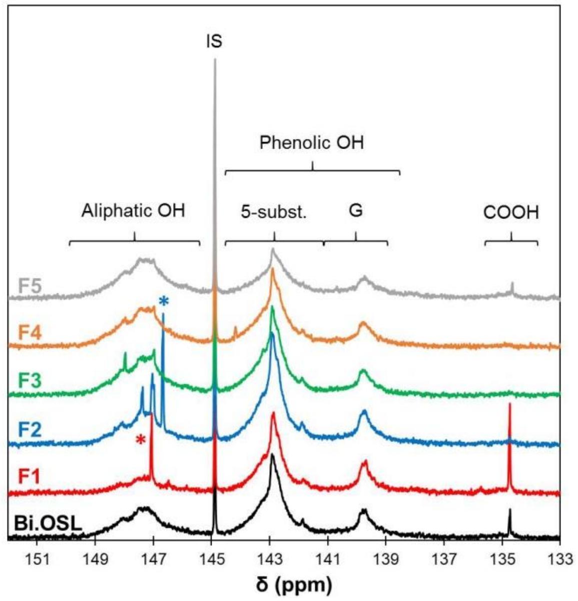

Figure S 8. ^{31}P NMR spectra of birch lignin and its fractions. Integration regions of the different functional groups were taken as described by Meng et al.[8] Peaks labeled with stars are residual peaks from solvents (1-propanol in F1 and ethanol in F2, respectively). Their integrals were deduced from the integrals of the aliphatic OH region to avoid an overestimation of lignin fractions content in aliphatic OH groups.

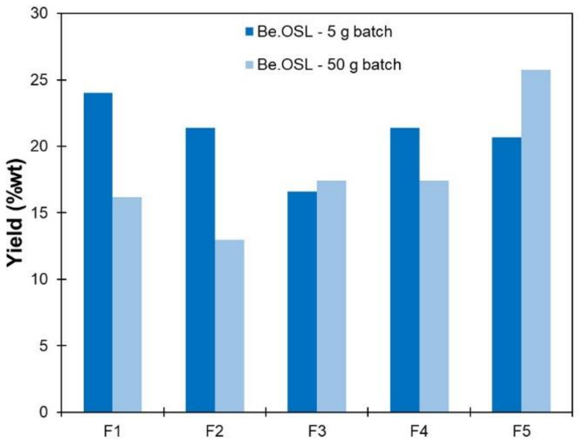

Figure S 9. Comparison of the yields of 2 distinct sequential solvent fractionations of beech lignin

Table S 5. Yields and average molar masses of the fractions obtained by two distinct sequential solvent fractionations of beech lignin performed at different scales

Scale	Fraction	Yield (%wt)	M_n (g mol^{-1})	M_w (g mol^{-1})	Đ
5 g	- [a]	-	1810	3610	1.99
	F1	24.0	1020	1470	1.44
	F2	21.4	1500	2070	1.38
	F3	16.6	2410	3360	1.39
	F4	21.4	3070	4650	1.51
	F5	20.7	4270	8460	1.98
50 g	- [a]	-	1810	3610	1.99
	F1	16.2	970	1410	1.45
	F2	12.9	1390	1940	1.40
	F3	17.4	2150	3200	1.49
	F4	17.4	2720	4480	1.65
	F5	25.8	3550	7520	2.12

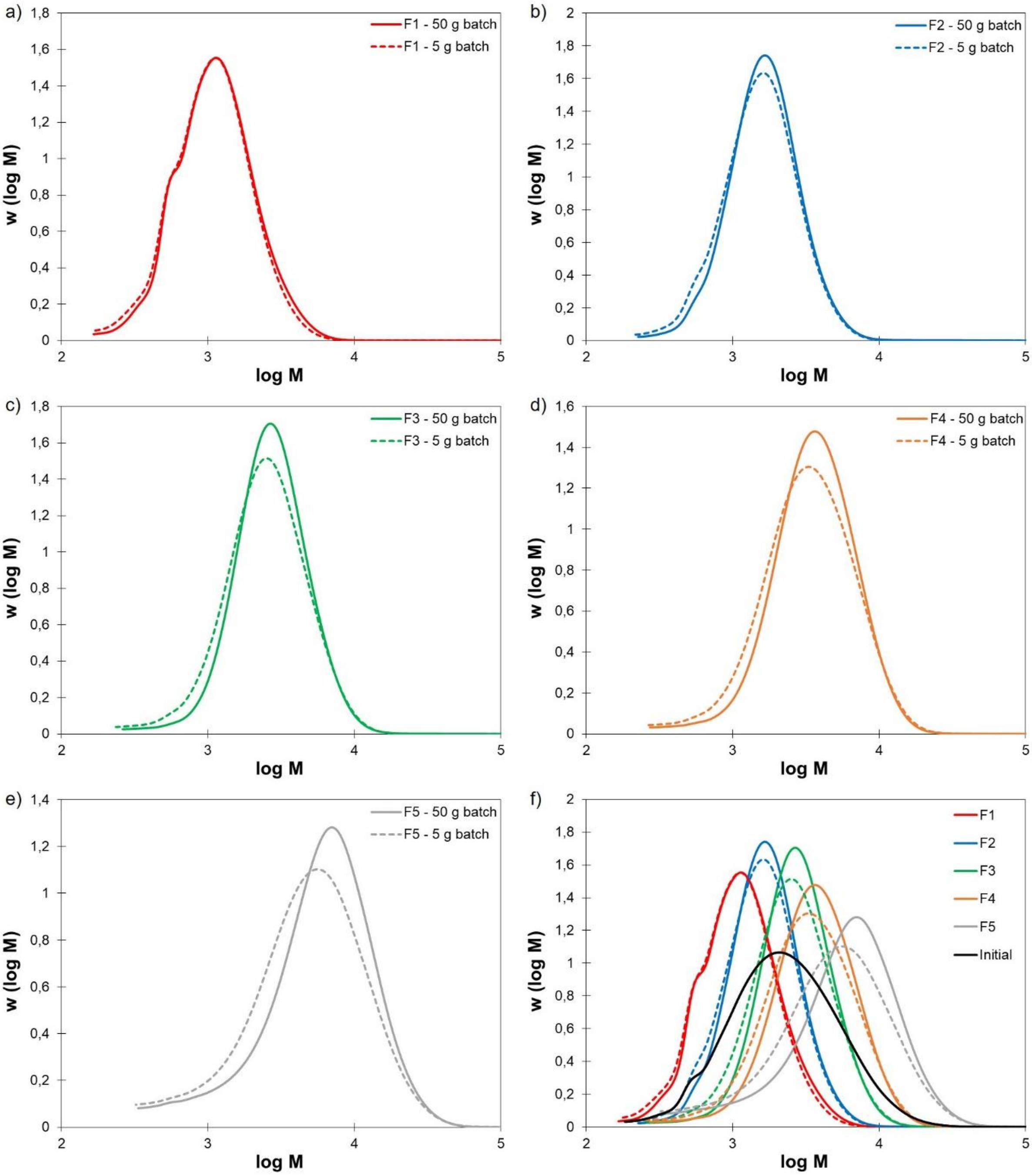

Figure S 10. Comparison of the SEC distributions of fractions of beech lignin obtained by 2 distinct sequential solvent fractionations: (a) F1, (b) F2, (c) F3, (d) F4, (e) F5, (f) overlay of the SEC distributions

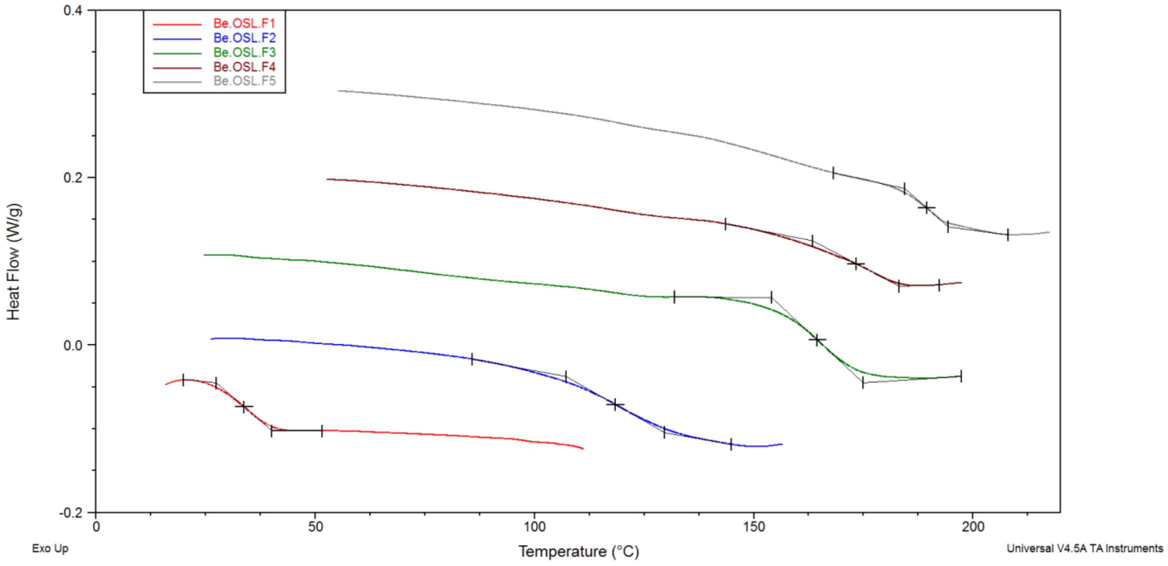

Figure S 11. DSC curves of beech lignin fractions

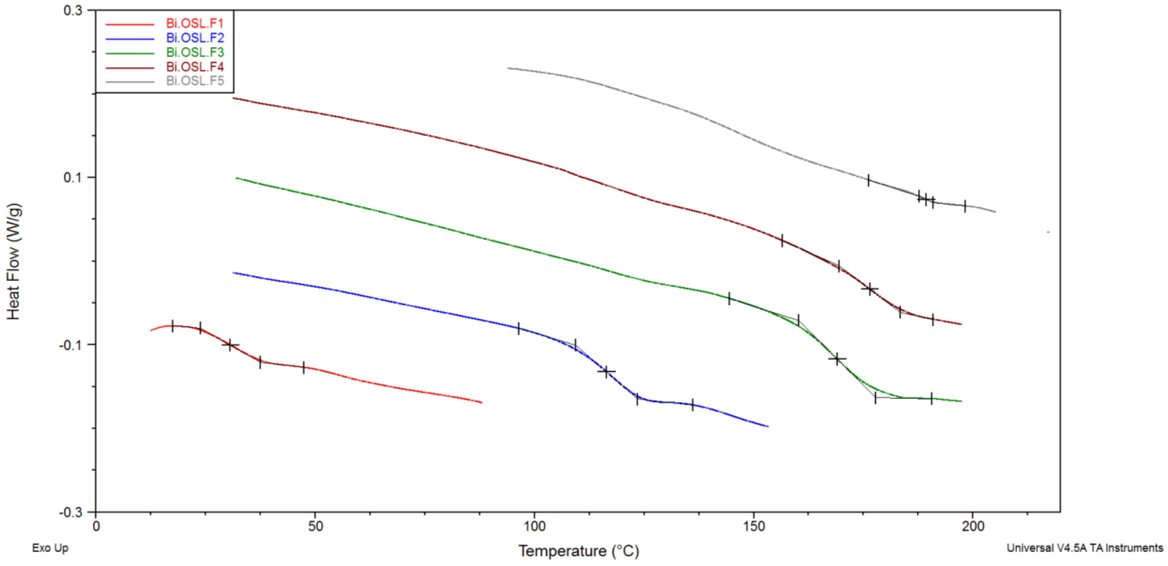

Figure S 12. DSC curves of birch lignin fractions

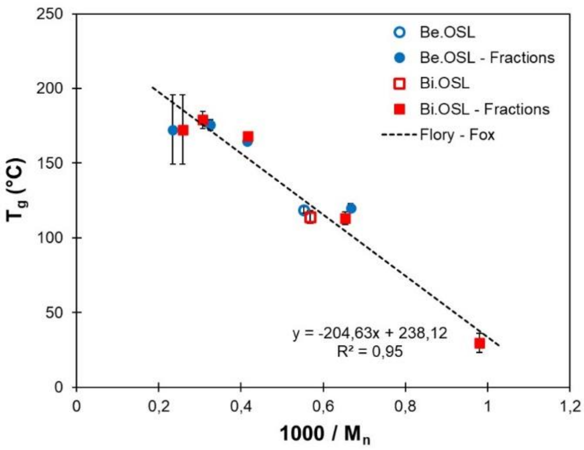

Figure S 13. Fit of the evolution of the glass transition of lignin fractions with the molar mass according to Flory-Fox relationship

Modification of lignins with ethylene carbonate (EC)

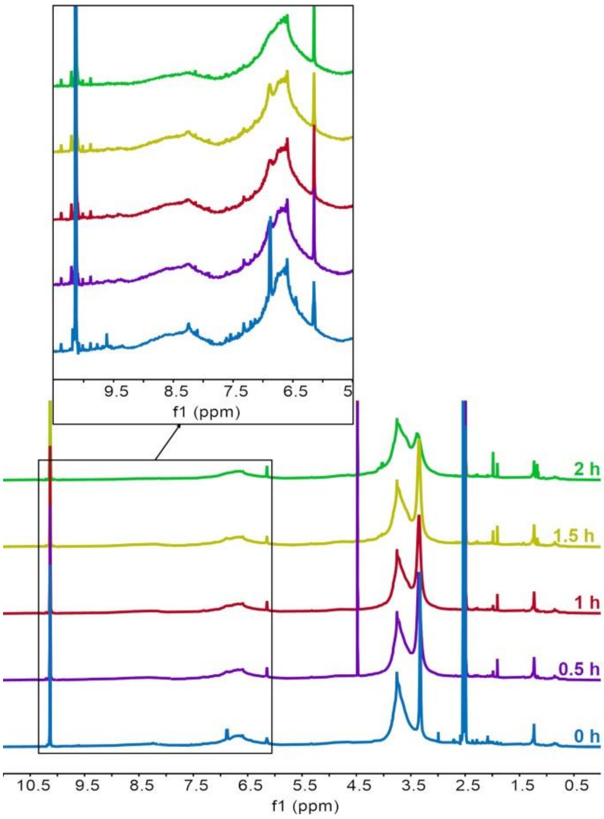

Figure S 14. ^{1}H NMR spectra (DMSO-d_6, 400 MHz) of beech lignin reacted with EC at 90 °C for different reaction times (10 eq. EC, 0.1 eq K_2CO_3). The inset shows the region of the phenolic OH protons (7.8 – 10 ppm).

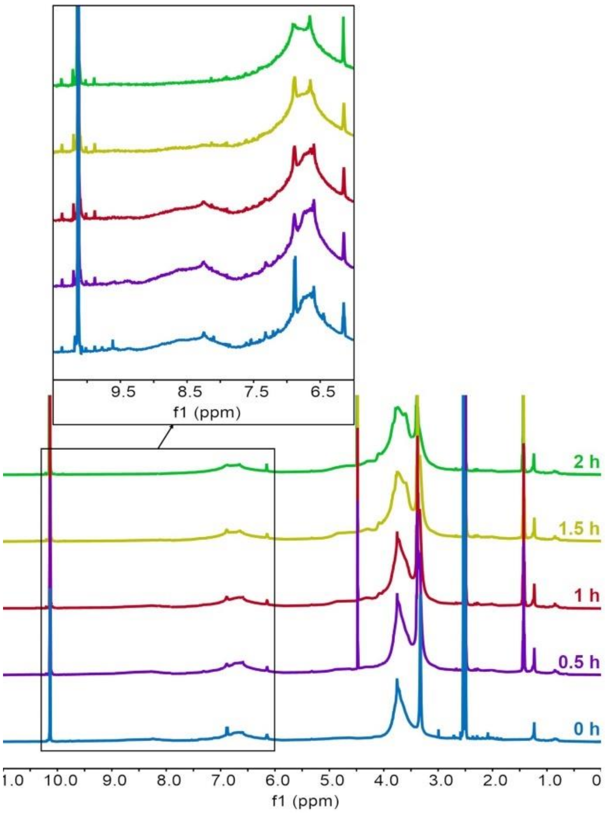

Figure S 15. ^{1}H NMR spectra (DMSO-d_6, 400 MHz) of beech lignin reacted with EC at 100 °C for different reaction times (10 eq. EC, 0.1 eq K_2CO_3). The inset shows the region of the phenolic OH protons (7.8 – 10 ppm).

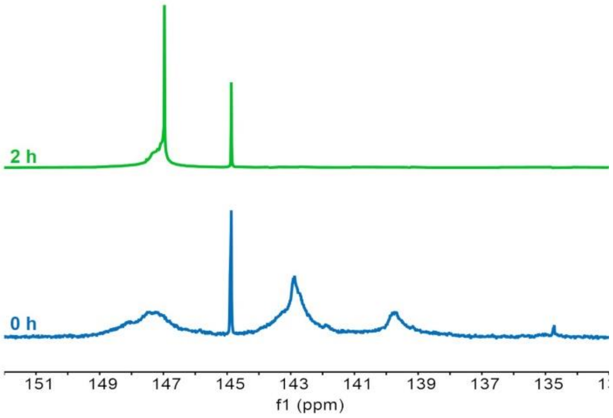

Figure S 16. ^{31}P NMR spectra of initial beech lignin and after reaction with EC at 100 °C for 2 h (10 eq. EC, 0.1 eq K_2CO_3)

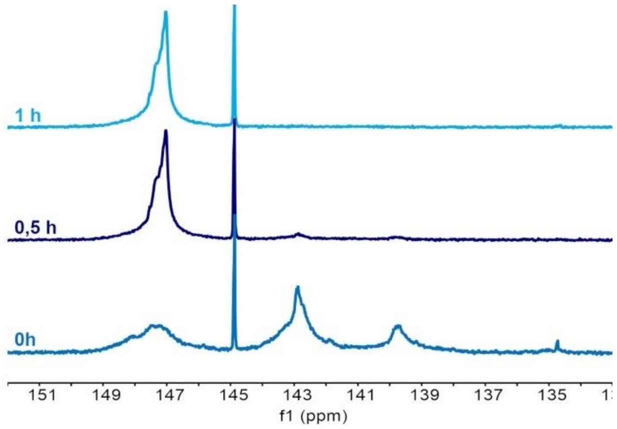

Figure S 17. ^{31}P NMR spectra of initial beech lignin and after reaction with EC at 110 °C for various reaction times (10 eq. EC, 0.1 eq K_2CO_3)

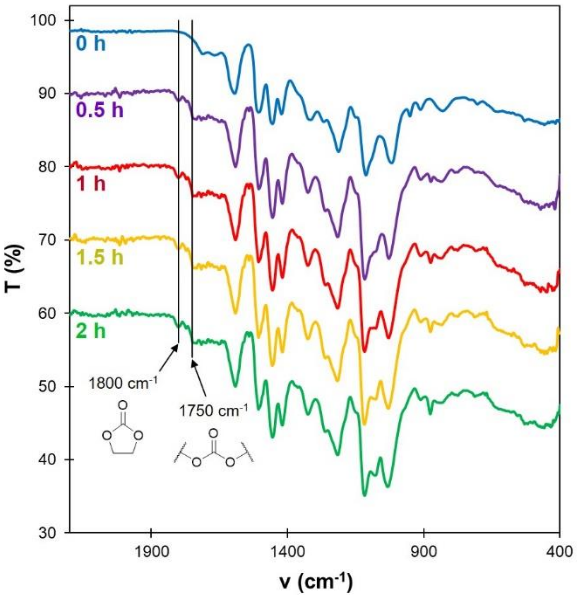

Figure S 18. FTIR spectra of beech lignin reacted with EC at 90 °C for different reaction times (10 eq. EC, 0.1 eq K_2CO_3). The small peak at 1800 cm^{-1} corresponds to C=O in unreacted EC which was not removed from the sample, the shoulder at 1750 cm^{-1} corresponds to C=O in linear carbonate linkages.

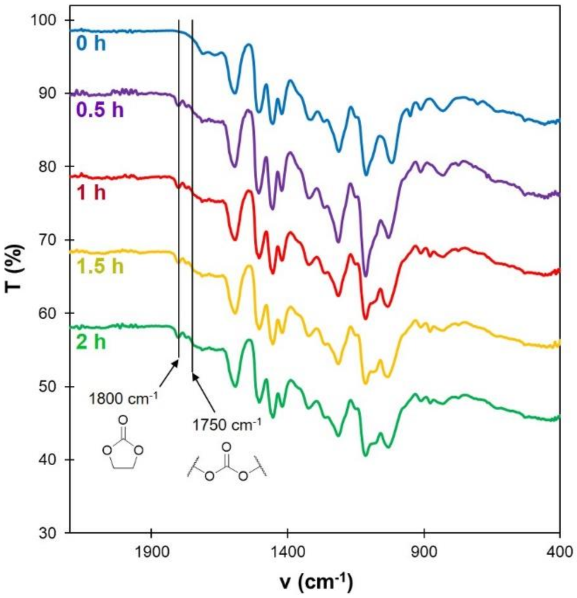

Figure S 19. FTIR spectra of beech lignin reacted with EC at 100 °C for different reaction times (10 eq. EC, 0.1 eq K_2CO_3). The small peak at 1800 cm^{-1} corresponds to C=O in unreacted EC which was not removed from the sample, the shoulder at 1750 cm^{-1} corresponds to C=O in linear carbonate linkages.

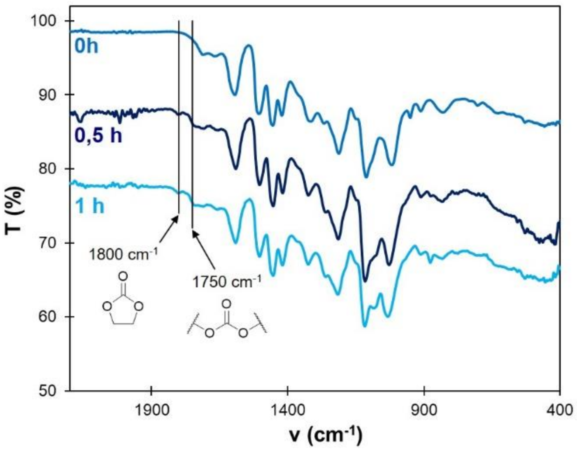

Figure S 20. FTIR spectra of initial beech lignin and after reaction with EC at 110 °C for various reaction times (10 eq. EC, 0.1 eq K_2CO_3). The small peak at 1800 cm^{-1} corresponds to C=O in unreacted EC which was not removed from the sample, the shoulder at 1750 cm^{-1} corresponds to C=O in linear carbonate linkages.

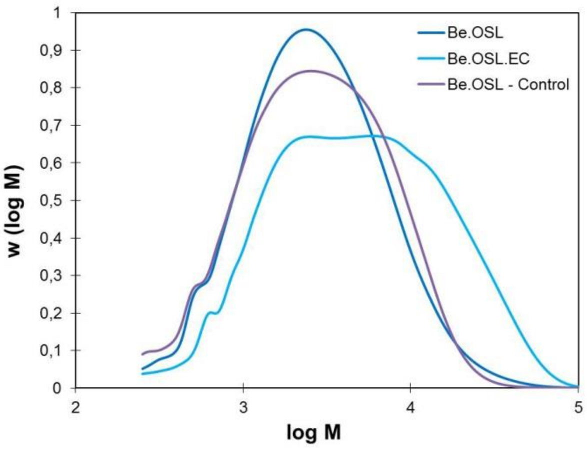

Figure S 21. Comparison of SEC distributions of the initial beech lignin, beech lignin after reaction with EC and beech lignin after a control reaction (blank) in DMF

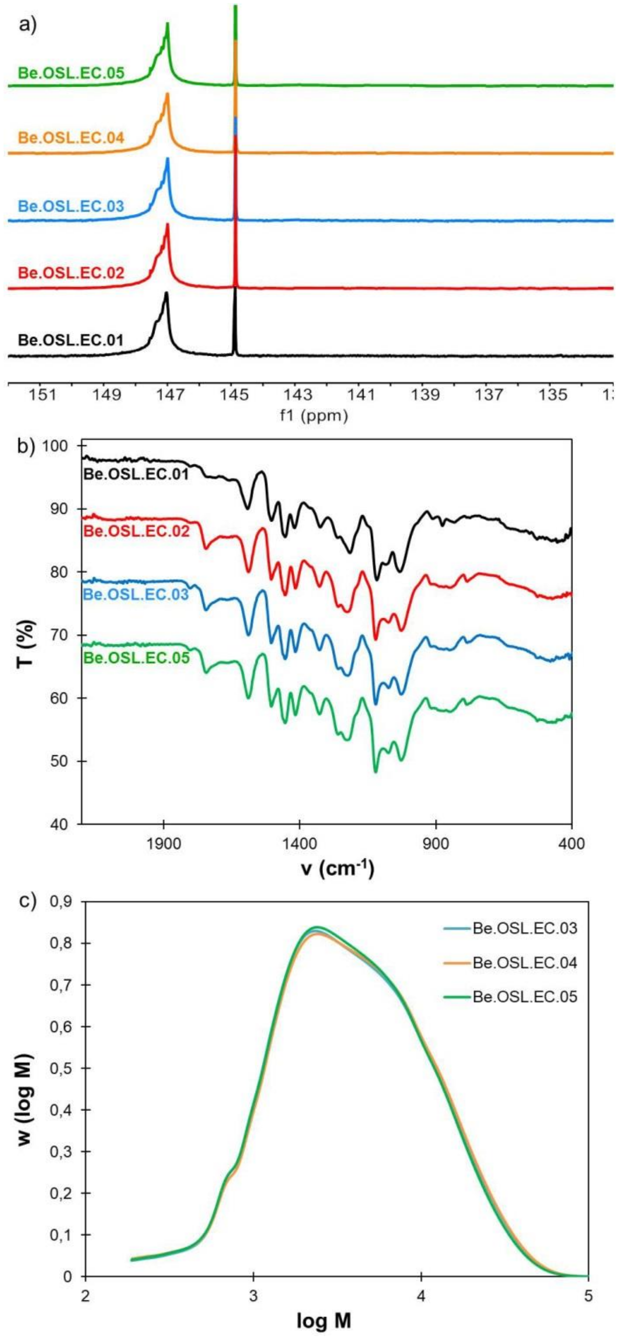

Figure S 22. Reproducibility assessment of the modification of beech lignin with EC (10 eq. EC, 0.1 eq. K_2CO_3, 110 °C, 1 h): (a) ^{31}P NMR spectra of 5 replicates of the reaction, (b) FTIR spectra of 4 replicates and (c) SEC distributions of 3 replicates

Table S 6. Reproducibility of the modification of beech lignin with EC (10 eq. EC, 0.1 eq. K_2C0_3, 110 °C, 1 h)

**Sample**	**Yield (%)**	**Ph-OH conv. (%)**	**Al-OH (mmol g^{-1})**	**Δ(Al-OH) (%)**	**M_n (g mol^{-1})**	**M_w (g mol^{-1})**	**Đ**
Be.OSL.EC.01	*na* [a]	100	3.18	68	- [b]	- [b]	- [b]
Be.OSL.EC.02	82	100	3.34	77	- [b]	- [b]	- [b]
Be.OSL.EC.03	81	100	3.32	76	2730	7500	2.75
Be.OSL.EC.04	80	100	3.28	74	2760	7620	2.76
Be.OSL.EC.05	82	100	3.45	83	2700	7270	2.69
Average	81 ± 1	100	3.31 ± 0.09	75 ± 5	2730 ± 30	7460 ± 150	2.73 ± 0.03

[a] *na* = not available, because aliquots were taken from the reaction mixture to follow the reaction by NMR

[b] not measured

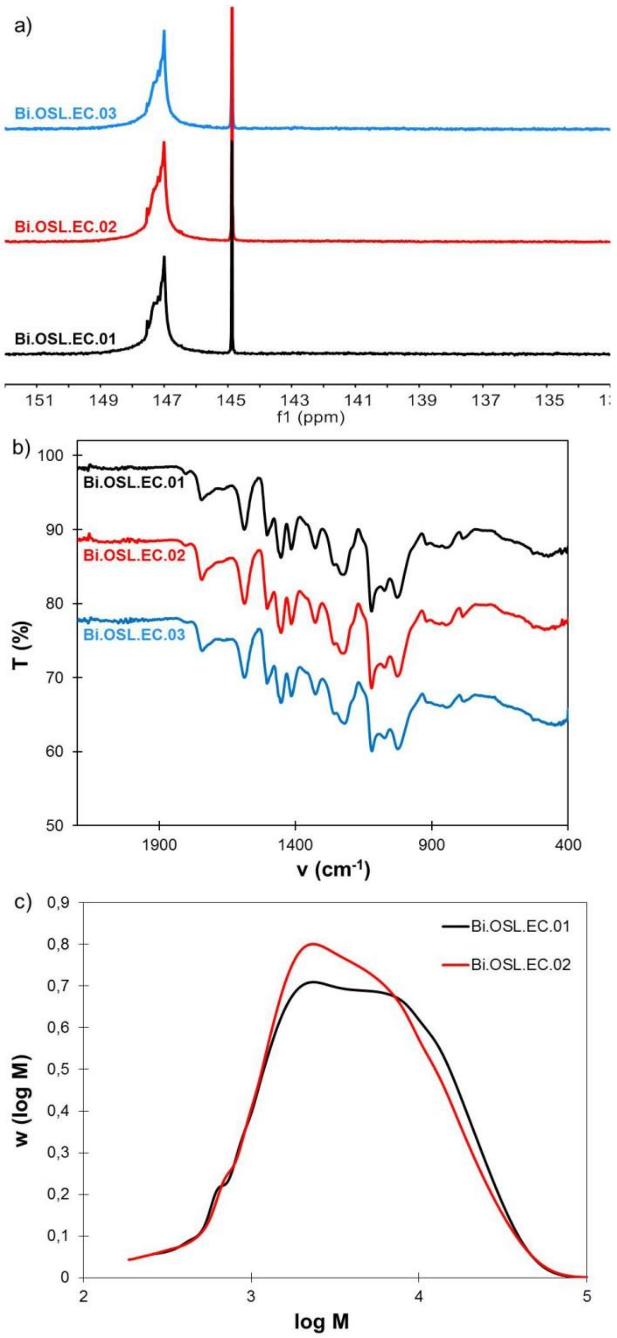

Figure S 23. Reproducibility assessment of the modification of birch lignin with EC (10 eq. EC, 0.1 eq. K_2CO_3, 110 °C, 1 h): (a) ^{31}P NMR spectra of 3 replicates of the reaction, (b) FTIR spectra of 3 replicates and (c) SEC distributions of 2 replicates

Table S 7. Reproducibility of the modification of birch lignin with EC (10 eq. EC, 0.1 eq. K_2C0_3, 110 °C, 1 h)

Sample	Yield (%)	Ph-OH conv. (%)	Al-OH (mmol g^{-1})	Δ(Al-OH) (%)	M_n (g mol^{-1})	M_w (g mol^{-1})	Đ
Bi.OSL.EC.01	*na* [a]	100	3.50	95	2620	8710	3.32
Bi.OSL.EC.02	*nm* [b]	100	3.06	70	2730	7930	2.90
Bi.OSL.EC.03	87	100	3.45	93	*nm* [b]	*nm* [b]	*nm* [b]
Average	87	100	3.34 ± 0.20	86 ± 11	2675 ± 55	8320 ± 390	3.11 0.21

[a] *na* = not available, because aliquots were taken from the reaction mixture to follow the reaction by NMR

[b] *nm* = not measured

Impact of lignin structure on its reactivity with ethylene carbonate

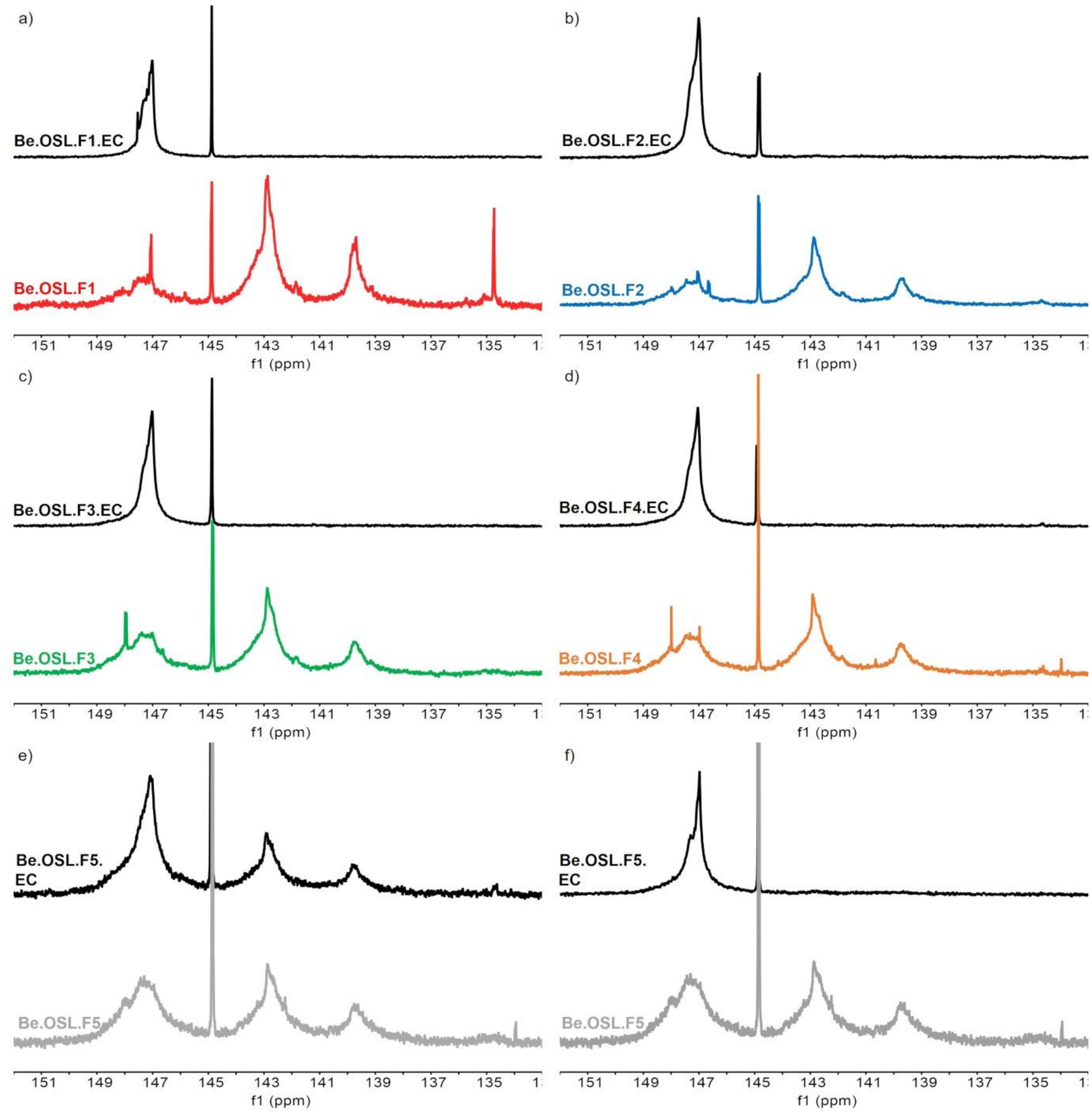

Figure S 24. ^{31}P NMR spectra of beech lignin fractions before and after reaction with EC (0.1 eq. K_2CO_3, 110 °C, 1 h): (a) F1 (10 eq. EC), (b) F2 (12 eq. EC), (c) F3 (16 eq. EC), (d) F4 (18 eq. EC), (e) F5 (27.5 eq. EC), (f) F5 (10 eq. EC in DMF as solvent)

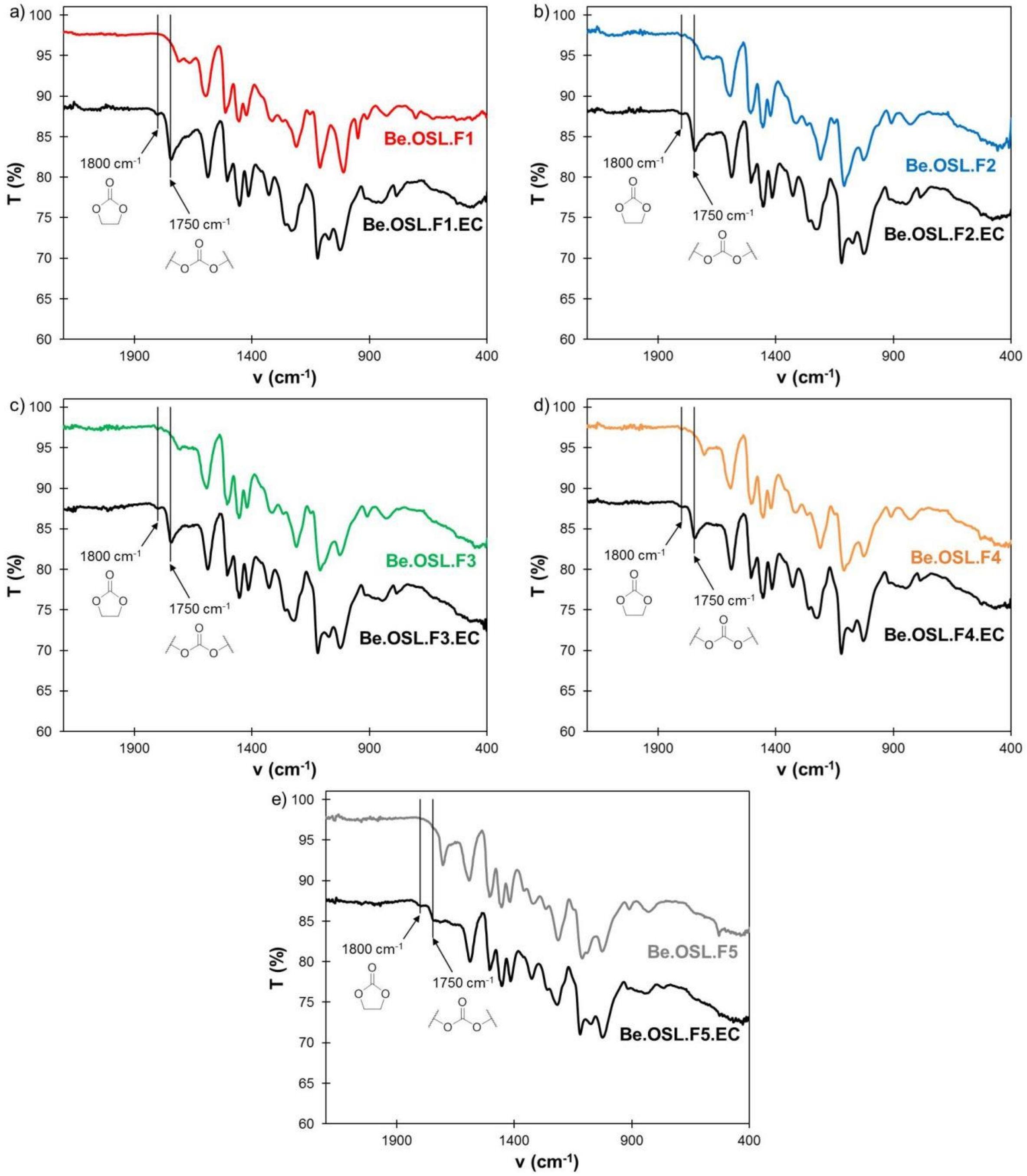

Figure S 25. FTIR spectra of beech lignin fractions before and after reaction with EC (0.1 eq. K_2CO_3, 110 °C, 1 h): (a) F1 (10 eq. EC), (b) F2 (12 eq. EC), (c) F3 (16 eq. EC), (d) F4 (18 eq. EC), (e) F5 (10 eq. EC in DMF as solvent)

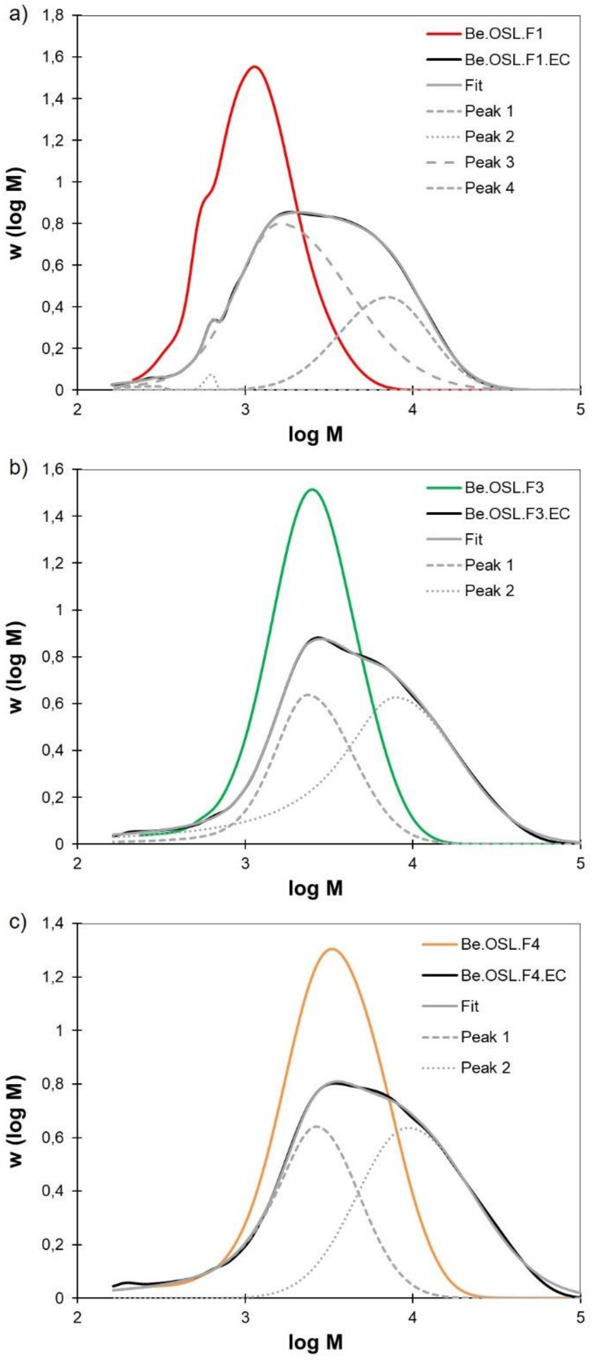

Figure S 26. Molar mass distributions of beech lignin fractions before and after reaction with EC: (a) F1, (b) F3), (c) F4. The molar mass distributions of the modified lignin fractions were deconvoluted with two peaks, except for F1 where 4 peaks were necessary to better represent the presence of low molar mass compounds.

References

[1] C. M. Hansen, *Hansen Solubility Parameters: A User's Handbook*, CRC Press, Boca Raton, FL, **2007**.
[2] M. J. Kamlet, J. L. M. Abboud, M. H. Abraham, R. W. Taft, *J. Org. Chem.* **1983**, *48*, 2877–2887.
[3] Y. Marcus, *Chem. Soc. Rev.* **1993**, *22*, 409–416.
[4] C. M. Hansen, A. Björkman, *Holzforschung* **1998**, *52*, 335–344.
[5] G. Cañete Vebber, P. Pranke, C. Nunes Pereira, *J. Appl. Polym. Sci.* **2014**, *131*, 39696.
[6] L. P. Novo, A. A. S. Curvelo, *Ind. Eng. Chem. Res.* **2019**, *58*, 14520–14527.
[7] A. Duval, F. Vilaplana, C. Crestini, M. Lawoko, *Holzforschung* **2016**, *70*, 11–20.
[8] X. Meng, C. Crestini, H. Ben, N. Hao, Y. Pu, A. J. Ragauskas, D. S. Argyropoulos, *Nat. Protoc.* **2019**, *14*, 2627–2647.
